# Multiple optimality criteria support Ornithoscelida

**DOI:** 10.1098/rsos.170833

**Published:** 2017-10-25

**Authors:** Luke A. Parry, Matthew G. Baron, Jakob Vinther

**Affiliations:** 1 Bristol Life Sciences Building, University of Bristol, 24 Tyndall Avenue, Bristol BS8 1TH, UK; 2 Department of Earth Sciences, Natural History Museum, London, Cromwell Road, London SW7 5BD, UK; 3 Department of Earth Science, University of Cambridge, Downing Street, Cambridge CB2 3EQ, UK

**Keywords:** Bayesian, phylogenetics, likelihood, Dinosauria, Avemetatarsalia, cladistics

## Abstract

A recent study of early dinosaur evolution using equal-weights parsimony recovered a scheme of dinosaur interrelationships and classification that differed from historical consensus in a single, but significant, respect; Ornithischia and Saurischia were not recovered as monophyletic sister-taxa, but rather Ornithischia and Theropoda formed a novel clade named Ornithoscelida. However, these analyses only used maximum parsimony, and numerous recent simulation studies have questioned the accuracy of parsimony under equal weights. Here, we provide additional support for this alternative hypothesis using Bayesian implementation of the Mkv model, as well as through number of additional parsimony analyses, including implied weighting. Using Bayesian inference and implied weighting, we recover the same fundamental topology for Dinosauria as the original study, with a monophyletic Ornithoscelida, demonstrating that the main suite of methods used in morphological phylogenetics recover this novel hypothesis. This result was further scrutinized through the systematic exclusion of different character sets. Novel characters from the original study (those not taken or adapted from previous phylogenetic studies) were found to be more important for resolving the relationships within Dinosauromorpha than the relationships within Dinosauria. Reanalysis of a modified version of the character matrix that supports the Ornithischia–Saurischia dichotomy under maximum parsimony also supports this hypothesis under implied weighting, but not under the Mkv model, with both Theropoda and Sauropodomorpha becoming paraphyletic with respect to Ornithischia.

## 1. Background and summary

Historically, dinosaurs have almost always been classified according to a dichotomic scheme, which was first proposed by Seeley [1], in which all dinosaurs are grouped into either Ornithischia or Saurischia, based primarily around the structure of their hip bones. This scheme has, for the most part, been recovered in modern phylogenetic analyses of dinosaurs [2–5]. However, a recent study [6] of early dinosaurs and other dinosauromorphs (close dinosaur relatives) recovered a tree topology within Dinosauria that differs radically from the historically accepted hypothesis of dinosaur evolution and interrelationships. The study by Baron *et al.* [6] produced this novel result by assembling and analysing the largest dataset of Triassic and Early Jurassic dinosauromorphs, assessing 457 anatomical characters in 75 taxa. The results in this initial study were produced using equal-weights parsimony, which has been most commonly applied for phylogenetic and evolutionary studies of extinct archosaurs [4,5,7].

The dataset of Baron *et al*. [6] was also analysed by Langer *et al*. [8] after they had made a substantial number of modifications to the taxon character scores. The analyses of Langer *et al*. [8] also only used equal-weights parsimony and the results of these analyses did not differ significantly from those of Baron *et al*. [6], although a traditional Saurischia–Ornithischia arrangement was found to be two steps shorter than the Ornithoscelida hypothesis.

There is a continuing debate concerning the optimality criterion for morphological data that is most appropriate and recovers the most accurate phylogeny. Typically, these debates have focused on parsimony with equal weights, implied-weights parsimony [9], and the Mk-likelihood model [10] in both maximum-likelihood and Bayesian implementations. This has recently been investigated in a number of simulation studies which have either favoured Bayesian inference [11–13] or implied-weights parsimony [14]. Analyses favouring Bayesian inference are robust to a number of perturbations such as model choice when generating the simulated matrices (particularly regarding violating some assumptions of the Mk model) [12,13], variations in tree symmetry [12], although see [14] and character evolutionary rate [11]. Analysis of empirical data supports the general conclusion that Bayesian Mk trees are more poorly resolved [12,15] and that matrices with few characters relative to the number of taxa or low phylogenetic signal may not be able to distinguish between competing phylogenetic hypotheses [12].

All of these studies generated their simulated matrices using models that assume that proportional branch lengths are shared by all characters, an assumption that may be inappropriate for morphological data [14]. When this assumption is relaxed, implied weighting is apparently favoured [14]. Which method performs best therefore appears to be contingent on the underlying assumptions of the simulations used to generate the character matrices, with Bayesian inference performing best when characters share common branch lengths [12,13]. Regardless of these outstanding methodological questions, all of the simulation studies outlined above favour other methods over parsimony with equal character weights. An exception are the analyses of Congreve & Lamsdell [16], which favoured equal weighting over implied weighting, but in analyses that used simulated matrices with an unrealistic distribution of homoplasy among characters [14].

It is clear that these competing phylogenetic methods produce profoundly different (or unresolved) trees [12] and therefore, in the absence of a consensus over which method performs best, topological incongruence between these methods obfuscates the relationships and evolutionary history of fossil taxa. Application of optimality criteria other than equal-weights parsimony to the dataset of Baron *et al.* [6] will therefore determine whether the recovery of Ornithoscelida from this dataset is robust to differing analytical conditions and will further investigate the impact of the character recodings proposed by Langer *et al.* [8].

## 2. Material and methods

Bayesian analyses were performed using MrBayes v. 3.2.6 [17] under the Mkv model, which corrects for the ascertainment bias [10], as only variable characters were scored in the character matrix. Rate variation among characters was modelled using four discrete gamma categories. The data were treated as a single partition, and analysis was performed where all characters were treated as ordered and also with characters ordered as in [6]. All Bayesian analyses ran for 10 000 000 generations, sampling every 1000 generations, with a burn-in fraction of 0.25. Convergence was assessed using average deviation of split frequencies (convergence at less than 0.01) and ESS (effective sample size) scores (convergence at ESS > 200) in MrBayes and graphically using Tracer v. 1.6, to ensure that the runs had reached stationarity prior to burn-in.

Analyses using implied-weights parsimony were carried out using the TNT 1.5-beta [18] using a range of concavity constants for implied weights (*k*-values); *k* was set at 3, 5, 10, 12, 15, 30, 50 and 60. For each value of *k*, trees were searched for using the New Technology Search function with ratchet set to 20 cycles until minimum tree length was hit 100 times. The MPTs found in this analysis were then subjected to a second round of TBR branch swapping. Both regular and extended implied weighting was used.

Finally, the distribution of phylogenetic signal among the included characters was investigated through a number of equal-weights parsimony and Bayesian analyses; the dataset being analysed was assembled by combining anatomical characters drawn from a number of phylogenetic studies of dinosaurs and other archosaurs [3,5,7,19] as well as a set of novel anatomical characters, the contribution of which are yet to be explored systematically. These characters were grouped into partitions, either based on position in the skeleton (cranial, postcranial and dental), or in terms of their origin (novel, taken/modified from previous studies) and excluded and included in different combinations to see what effect, if any, the removal of such sets would have on the tree topologies recovered. A Bayesian analysis was also carried out on a partitioned dataset, in which the non-dental cranial characters, the dental characters and the postcranial characters had their own substitution model.

## 3. Results

**Figure 1.**
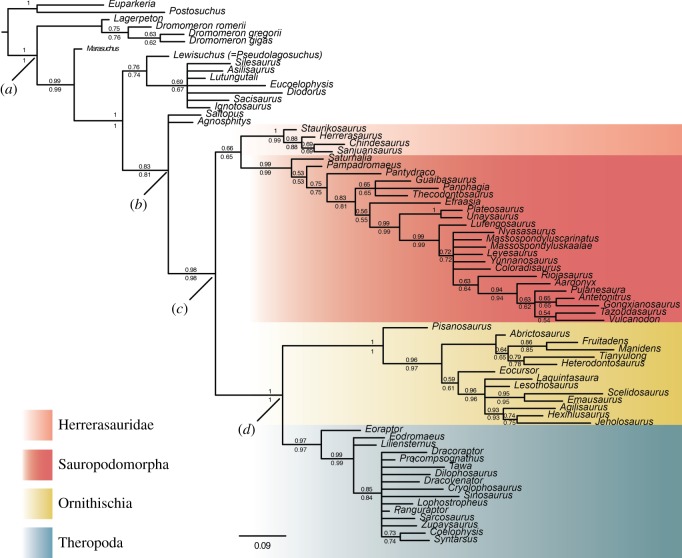
Results of the Bayesian analyses. Numbers at nodes are posterior probabilities shown to two significant digits with analyses which treated certain characters as ordered shown below and no characters as ordered above. The scale bar is in expected number of substitutions per site. Labelled clades are (*a*) Dinosauromorpha, (*b*) unnamed clade (Dinosauria + *Saltopus* + *Agnosphitys*), (*c*) Dinosauria and (*d*) Ornithoscelida.

The results of the Bayesian analyses under the Mkv model support the topology originally recovered by Baron *et al.* [6] (figure 1) when using the entire dataset. Dinosauria is monophyletic and is divided into Saurischia (Sauropodomorpha and Herrerasauridae) and Ornithoscelida (Ornithischia and Theropoda). The deep nodes which define major clades (e.g. Dinosauromorpha, Dinosauria, Ornithoscelida) are well supported (PP > 0.9). Support values for the different Bayesian analyses for the major groups are summarized in table 1.

**Table 1.** Summary of posterior probabilities from Bayesian analyses under the Mkv + gamma model in MrBayes. Analyses using only dental, only braincase and no postcranial characters.

clade	unordered	with ordered characters	no novel characters	partitioned	dental excluded	no skull/ braincase	only postcranial
Dinosauria	0.98	0.98	0.98	0.96	0.98	0.77	0.5
Herrerasauridae	1	0.99	0.98	1	1	0.98	—
Sauropodomorpha	0.99	0.99	0.97	0.98	0.97	0.98	—
Ornithischia	1	1	1	1	0.97	1	—
Theropoda	0.97	0.97	0.91	0.89	0.97	—	—
Saltopus/Agnophitys/Dinosauria	0.83	0.81	0.71	0.92	0.77	0.74	0.72
Ornithoscelida	1	1	0.98	1	1	0.78	—
Herrerasauridae/Sauropodomorph	0.66	0.65	—	0.67	0.82	—	—
Sauropodomorph/Ornithoscelida	—	—	0.6	—	—	0.5	—

The only ways in which the tree that was recovered using the Mkv model differs from the tree presented by Baron *et al.* [6] are reduced resolution within Neotheropoda and in the position of the enigmatic European dinosauriform *Agnosphitys cromhallensis*. In the original study, *Agnosphitys* was recovered within the dinosauriform clade Silesauridae [6] but in our results it falls outside of this clade, along with another European taxon, *Saltopus elginensis*. These taxa form a polytomy with Dinosauria. In this sense, the tree also differs from that of Baron *et al.* [6]; the position of *Saltopus* as a taxon forming part of a sister group to Dinosauria is not as well supported in our results. A similar position has been suggested for *Nyasasaurus parringtoni* before [12,20] but our results match those of Baron *et al.* [6], with *Nyasasaurus* being recovered within Sauropodomorpha, close to massospondylids such as *Massoposndylus carinatus.* This position for *Nyasasaurus* is supported by a number of features, including the possession of an ‘insertion’ vertebrae positioned between the first and second primordial sacrals; dorsoventrally tall sacral ribs; hyposphene-hypantrum intervertebral articulations in the presacral column; and a well-developed and proximodistally extensive deltopectoral crest that continuous with the proximal surface of the humerus and is laterally deflected at its tip.

**Figure 2.**
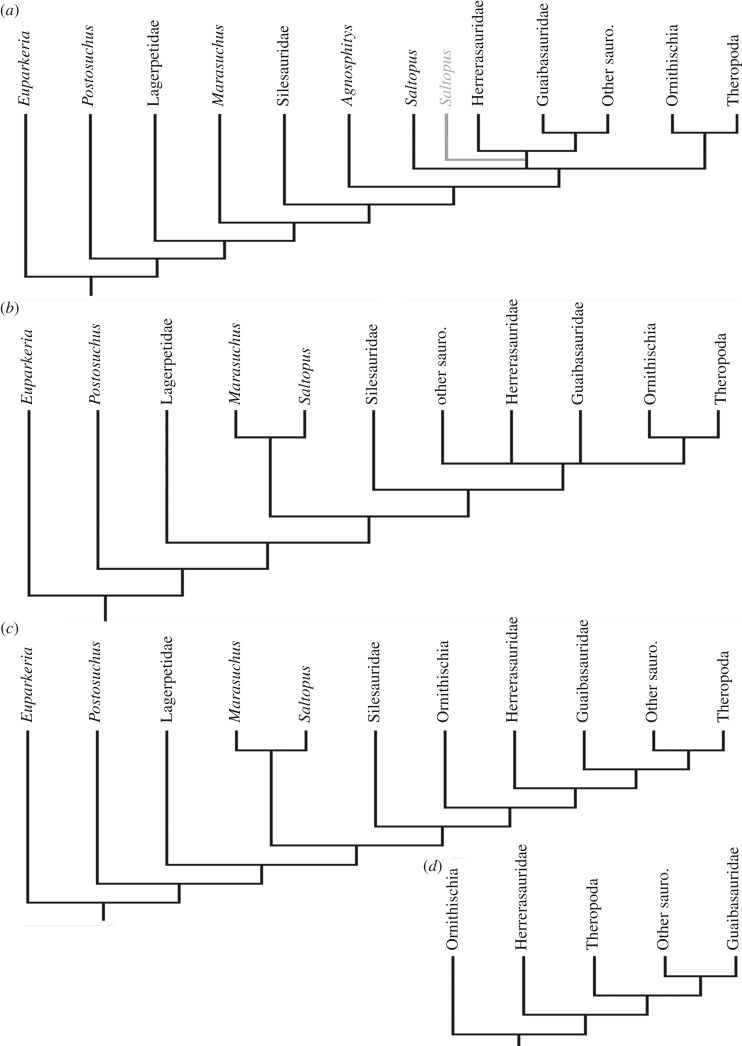
Simplified version of the strict consensus trees produced from the results of the implied-weights parsimony analyses. For each value of *k*, an analysis was run and a strict consensus tree was produced from the trees found in each search. The strict consensus trees produced for each value of *k* were then compared and a consensus of those trees was produced (black). The position of *Saltopus* within Saurischia was found for all values of *k* apart from 50 (grey) when using the original dataset of Baron *et al*. [6]. (*a*) Results obtained using the original dataset of Baron *et al*. [6]; (*b*) results obtained when using the dataset as modified by Langer *et al*. [8], for *k* = 3; (*c*) for 3 < *k* < 15 values less than 15 and (*d*) for *k*-values of 15 or more.

Using implied weighting, the strict consensus trees that were produced for each value of *k* only differ from each other in very minor ways, with the fundamental topology being consistent throughout the various results. A strict consensus of these trees recovers a monophyletic Dinosauria, Ornithoscelida and Saurischia, as redefined by Baron *et al.* [6] (figure 2).

**Figure 3.**
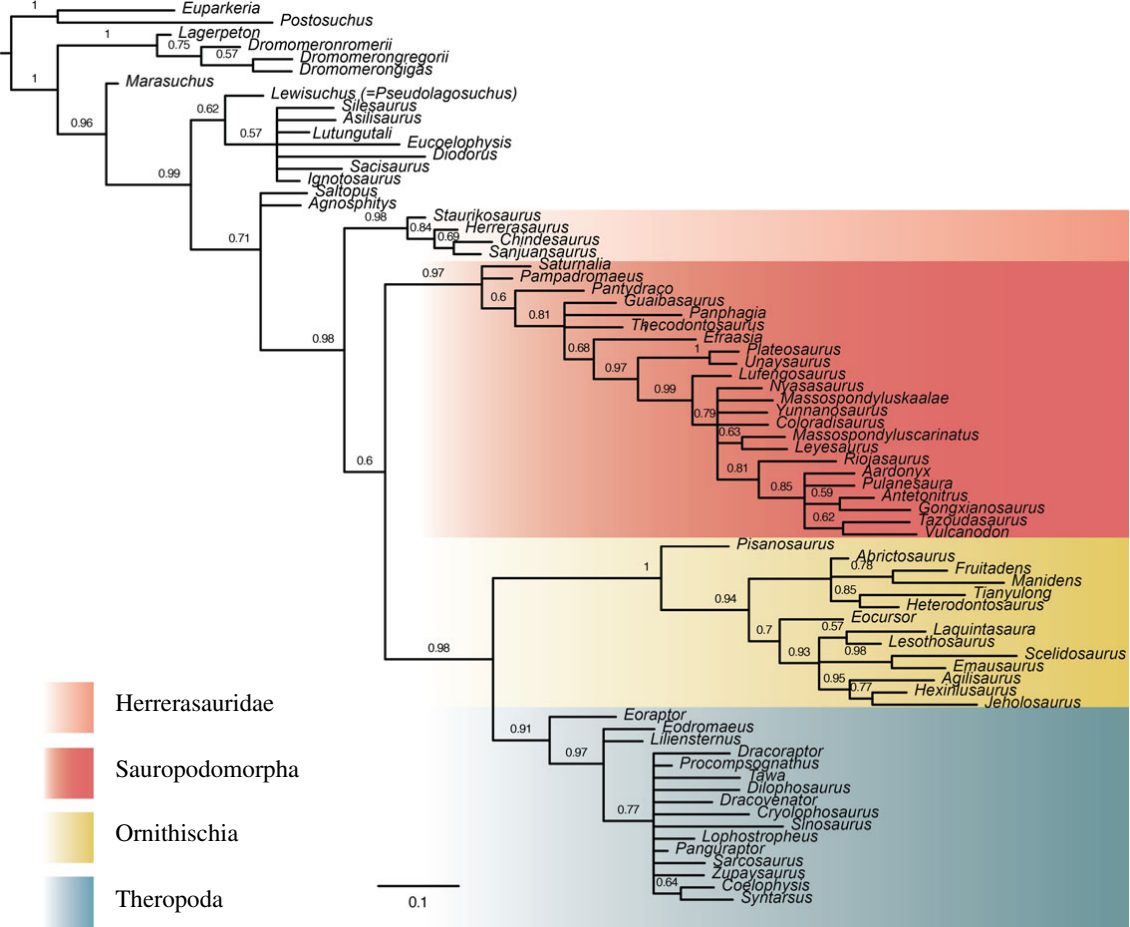
Results of the Bayesian analysis with no novel characters. This tree was produced from a reduced dataset that did not include the novel characters proposed by Baron *et al*. Here, Ornithoscelida is recovered as monophyletic, but Saurischia paraphyletic, with Herrerasauridae falling outside of Dinosauria. Numbers at nodes are posterior probabilities shown to two significant digits. The scale bar is in expected number of substitutions per site.

By excluding the novel anatomical characters by Baron *et al.* [6], Dinosauria is recovered in a polytomy with many dinosauriforms, including *Agnosphitys* and *Saltopus*, and Lagerpetidae, in the parsimony analysis (see the electronic supplementary material). The Bayesian analysis that excluded the novel character set also recovered a monophyletic Ornithoscelida and provided better resolution within Dinosauromorpha (figure 3). Interestingly, this analysis places Herrerasauridae outside of Saurischia, as recently redefined [6], becoming the sister group of Dinosauria. That the position of Herrerasauridae changes following a minor perturbation of the character data is perhaps not surprising given that in analyses of the whole dataset using Bayesian inference the node support for the sauropodomorph--herrerasaurid clade is low (approx. 0.6), when compared with the nodes defining the other higher clades of dinosaurs.

Parsimony and Bayesian analyses of individual character sets were largely unable to resolve the relationships of the taxa analysed; analyses of only cranial characters, only dental characters and only characters of the skull and brain case were unable to resolve any high order relationships within Archosauria (see the electronic supplementary material). We performed analyses on six subsampled and partition datasets, which either excluded or only included data from dental characters, non-dental cranial characters or characters of the postcranium. These analyses varied in character number from 36 to 422, in proportion of missing data from 0.42 to 0.61 and resolution from 2 to 44 nodes. Table 2 summarizes the proportion of missing data and the absolute resolution (in number of nodes) of these analyses. Resolution strongly positively correlates with number of characters contained in a partition (Pearson's = 0.95, *p* = 0.001) and that there is no strong correlation between the proportion of missing data and tree resolution (Pearson's = −0.01, *p* = 0.85).

**Table 2.** Distribution of missing data and resolution under analyses of different character partitions. Resolution is expressed in number of nodes.

character partition	no. characters	proportion missing	resolution
all	458	0.56	47
cranial	149	0.71	4
postcranial	273	0.55	26
dental	36	0.42	2
excluding cranial	309	0.48	36
excluding postcranial	185	0.57	5
excluding dental	422	0.57	44

**Figure 4.**
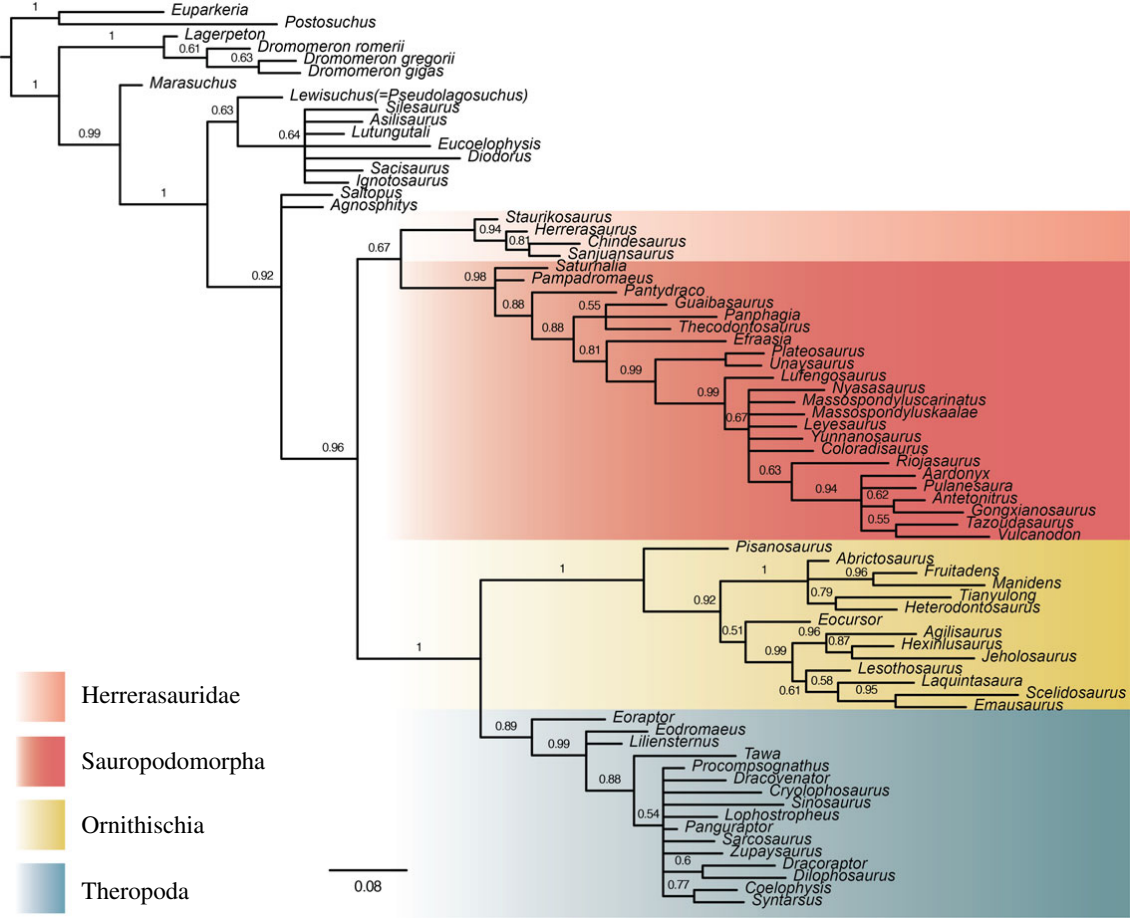
Results of the partitioned Bayesian analysis in which non-dental cranial, dental and postcranial characters had separate substitution models. Numbers at nodes are posterior probabilities shown to two significant digits. The scale bar is in expected number of substitutions per site.

Parsimony analysis of the postcranial character set recovered a polytomy containing most dinosaurs and other dinosauriforms, but, with the removal of four wildcard taxa, a tree topology resembling that of the original analysis is recovered, including a monophyletic Ornithoscelida (see the electronic supplementary material). Partitioned Bayesian analysis of the dataset, in which each character set had its own substitution model, produced better resolution with Archosauria and Dinosauromorpha, and recovered the same tree topology as the non-partitioned analyses, with a monophyletic Ornithoscelida (figure 4).

Implied-weights parsimony, when applied to the dataset of Langer *et al*. [8], produced a range of topologies (figure 2). For all *k*-values above 3, Ornithischia was consistently recovered outside of the clade containing all other dinosaurs (i.e. Sauirschia). However, for a *k*-value of 3, a monophyletic Ornithoscelida was recovered using the dataset as modified by Langer *et al*. [8] (figure 2). The ingroup relationships within Saurischia differed between the various implied-weights parsimony analyses: for all *k*-values below 15 the ‘guaibasaurids’ were recovered outside of the Theropoda–Sauropodomorpha clade (i.e. Eusaurischia), whereas as for all *k*-values of 15 and above, the ‘guaibasaurids’ were recovered within Sauropodomorpha. Herrerasauridae was consistently recovered outside of Eusaurischia in all of the analyses. *Saltopus* was found to fall outside of Dinosauromorpha in the analyses which set *k* at 3; for all *k*-values above 3 *Saltopus* was recovered within Dinosauromorpha and Dinosauriformes, as the sister-taxon to *Marasuchus* (figure 2).

**Figure 5.**
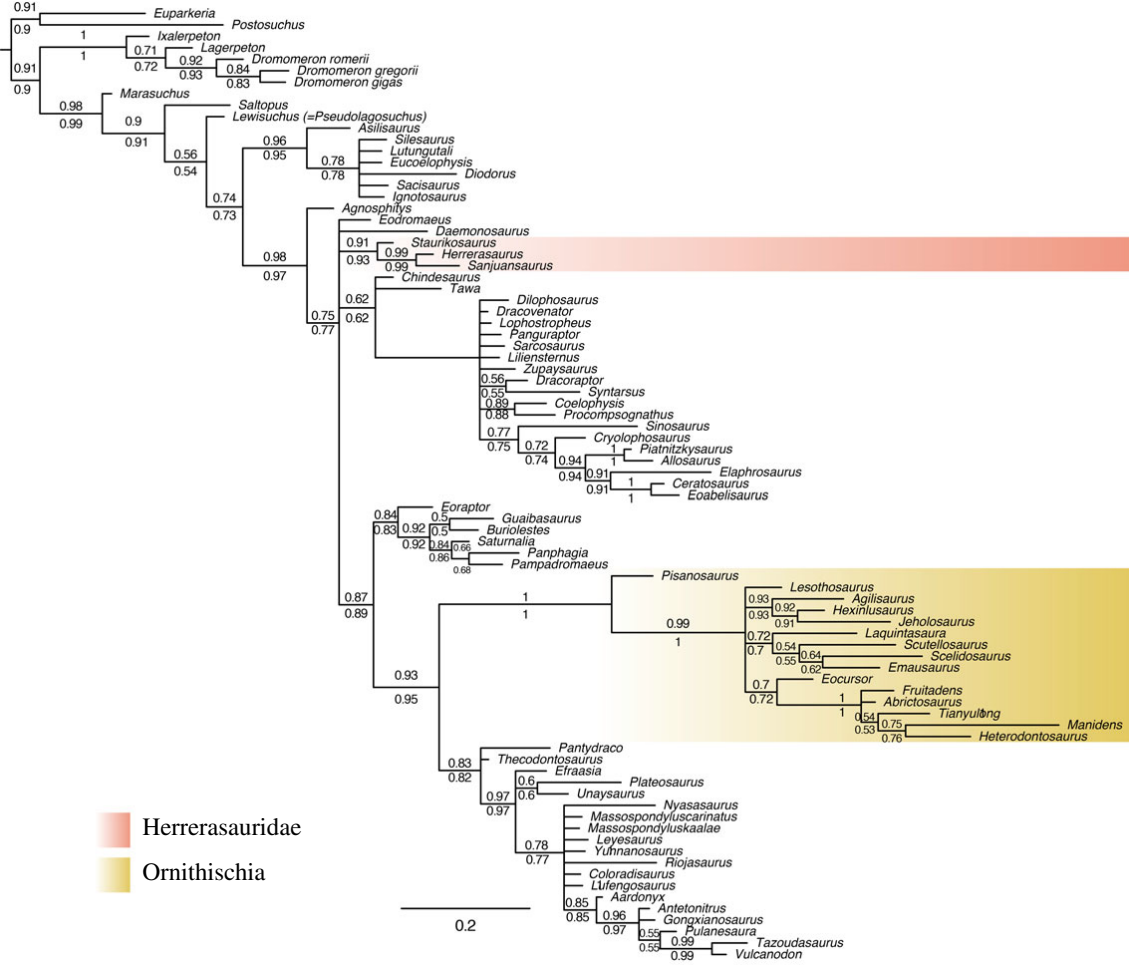
Results of the Bayesian analysis of the rescored dataset produced by Langer *et al*. [8], in which Theropoda and Sauropodomorpha are found to be paraphyletic. Numbers at nodes are posterior probabilities with values for no ordered characters and ordered characters above and below branches, respectively. The scale bar is in expected number of substitutions per site.

Bayesian analysis of the rescored dataset recovers a different set of topologies than were found by both the analyses of the original dataset and the equal- and implied-weights parsimony analyses of the rescored dataset (figure 5). This analysis differs from parsimony analyses in that only Herrerasauridae and Ornithischia are recovered as monophyletic with Theropoda and Sauropodomorpha becoming paraphyletic. However, the membership of Herrerasauridae is slightly different, with *Chindesaurus* nesting within Theropoda, in a polytomy with *Tawa* and Neotheropoda. Additionally, *Guaibasaurus* falls within Gauibasauridae in the results of the Bayesian analysis, unlike in the results of the equal-weights parsimony analysis presented by Langer *et al*. [8].

## 4. Discussion

A monophyletic Ornithoscelida was recovered in each of the analyses of the original dataset [6] where dinosaur relationships could be resolved, including both parsimony and Bayesian analyses, suggesting that this arrangement of the major clades within Dinosauria is well supported by the current data.

Although its utility in analysing morphological datasets has been debated [16], implied-weights parsimony has been treated by many previous studies as a ‘stress-test’ for the robustness of phylogenetic results under parsimony [16,21] and it may outperform other methods in particular circumstances [14]. Our implied-weights analyses support the tree topology recovered by Baron *et al.* [6] and recover similar topologies within each clade as the equal-weights parsimony and Bayesian analyses. The phylogenetic methods employed herein can produce topologies with substantial conflict from the same dataset [12], and so the fact that the fundamental relationships of the included taxa using alternative methods to equal weighting do not change further supports the hypothesis of Baron *et al.* [6].

The results of our Bayesian and equal-weights parsimony analyses differed when we used reduced datasets, notably when the novel anatomical characters presented by Baron *et al.* [6] were removed. Ornithoscelida was again recovered by both analyses, but the topology within dinosauriformes differed between the analyses in terms of resolution and the position of Herrerasauridae. This difference in results suggests that phylogenetic signal supporting Ornithoscelida is still present in this reduced dataset (i.e. without novel characters) and that the polytomy recovered in the first analysis may be an artefact of parsimony analysis.

Partitions in morphological data may contain varying or conflicting phylogenetic signal as a result of variations in structural complexity, functional constraints or convergent evolution in ecologically similar but phylogenetically distant taxa and also as a result of researcher bias when coding morphological characters [22,23]. For instance, dental characters can often convey different phylogenetic signals from osteological characters and may be less reliable for phylogenetic reconstructions. This phenomenon is particularly pronounced in mammals [23] but has also been demonstrated for vertebrates more generally, with significantly conflicting topologies found by dental and skeletal characters in a meta-analysis of vertebrates using parsimony [22]. By excluding only those characters in the dataset that are related to dentition and analysing datasets lacking craniodental characters entirely, such a difference in signal between partitions could be assessed.

When we performed character set exclusion experiments under equal-weights parsimony, the same fundamental tree structure, with the Ornithoscelida–Saurischia dichotomy within Dinosauria, was recovered (see the electronic supplementary material). In a previous meta-analysis of differing signal between the cranium and postcranium under parsimony, cranial characters were found to outnumber postcranial characters [22], an observation that does not hold true for the data analysed herein, where there were 273 and 185 characters for these partitions, respectively. In this meta-analysis, incongruent trees were found by analysing each partition separately in about a third of cases, a pitfall that may have been avoided in the dataset of Baron *et al*. [6] by scoring extensively from the postcranial skeleton.

By contrast, Bayesian analyses of individual character partitions were largely unable to resolve the deep relationships of the taxa considered. Only the postcranial characters recovered the monophyly of Dinosauria from Bayesian inference, albeit at the expense of almost all resolution. This analysis resolved only 26 nodes in the majority rule consensus compared with the 47 nodes recovered from all characters (table 2 and electronic supplementary material) and failed to recover the monophyly of Herrerasauridae, Sauropodomorpha, Ornithischia and Theropoda. Craniodental characters were also unable to resolve the phylogeny, recovering only five nodes. If such a lack of resolution from using the Mkv model turns out to be widespread for craniodental characters, then it is possible that the topological incongruence observed by Mounce *et al*. [22] resulted from over-resolution of consensus trees through use of parsimony on datasets unable to discriminate between alternative topologies. In this regard, our results support the general conclusion of Mounce *et al*. [22] that character data should be scored as extensively as possible, especially when using Bayesian inference.

The dental partition was most complete (42% missing) and the cranial partition was least complete (71% missing) but both partitions recovered a similar (and small) number of nodes, two and four, respectively, further suggesting that missing data were not driving the lack of resolution from individual partitions. This is congruent with other empirical analyses of morphological datasets using Bayesian inference under the Mkv model, where small character matrices cannot adequately resolve relationships [12]. This is further corroborated by the strong positive correlation between absolute resolution and number of characters (see Results). The enhanced completeness of dental data relative to other partitions matches the expectation that teeth have elevated preservation potential compared to bones.

The fact that a monophyletic Ornithoscelida is recovered with and without the inclusion of sets of characters under parsimony indicates the phylogenetic signal supporting Ornithoscelida is present across a range of anatomical ‘zones’ in the taxa considered, but Bayesian analysis suggests that these ‘zones’ in isolation lack the discriminatory power to resolve the phylogeny. The recovery of Ornithoscelida excluding novel characters suggests that existing data could have been used to challenge the historic hypothesis of dinosaur evolution had it been applied to a broader range of dinosaur taxa.

The novel anatomical characters scored by Baron *et al.* [6] add further resolution within Dinosauromorpha. Under implied-weights parsimony, a position for *Saltopus* as the sister group of Dinosauria is proposed, although it must be noted that implied weighting may over-resolve nodes with low support and that this position is not strongly supported by the current data. In our analyses, we recover *Saltopus* either as the sister group of Dinosauria or along with *Agnosphitys* in a polytomy using the Mkv model. This suggests that these taxa may be important for our understanding of early dinosaur evolution, particularly if their precise relationships can be clarified. While *Saltopus* exhibits the plesiomorphic dinosauriform condition of possessing only two sacral vertebrae, it also appears to possess a number of more derived dinosaurian features, including a straight, rod-like pubis and a tibia that is longer than the femur.

The results generated using the dataset of Langer *et al*. [8] differ from one another depending on the type of analysis used. Using equal- and implied-weights parsimony, something similar to a traditional Sauirschia–Ornithischia scheme is recovered fairly consistently. However, under Bayesian analysis an entirely new topology is recovered with many previously recognized clades becoming paraphyletic. While these results suggest that the recovery of Ornithoscelida is contingent on the preferred interpretation of some characters, it highlights that alternative interpretations do not necessarily support the Sauirschia–Ornithischia hypothesis.

## 5. Conclusion

Phylogenetic hypotheses generated using morphological data are sensitive to a number of variables including character sampling, the optimality criterion and its implementation. Our results show that the Ornithoscelida–Saurischia hypothesis (i.e. Pachypodosauria–Ornithoscelida hypothesis [24]) of dinosaur phylogeny is recovered using a range of methods, including implementations of parsimony and Bayesian inference using the Mkv model. Character exclusion experiments demonstrate that the Ornithoscelida–Saurischia hypothesis is robust to numerous perturbations of the underlying character data, and only collapses to a polytomy (rather than recovering an alternative topology) when only subsets of the available data are analysed under Bayesian inference. Minor permutations of the character data such as excluding particular character partitions influence resolution most strongly and also change the position of herrerasaurids relative to the other major clades of dinosaurs in some analyses. Reinterpretations of the character codings of Baron *et al*. [6], as proposed by Langer *et al*. [8], do not unequivocally support the Saurischia–Ornithischia hypothesis, as these character rescorings recover a topology under the Mkv model that is at odds with all previous phylogenies of dinosaurs.

## Supplementary Material

Support for Ornithoscelida: additional information on the methods and results.

## Data accessibility

The datasets supporting this article have been uploaded as part of the electronic supplementary material.
