# Correction to ‘Multiple optimality criteria support Ornithoscelida’

**DOI:** 10.1098/rsos.180154

**Published:** 2018-03-14

**Authors:** L. A. Parry, M. G. Baron, J. Vinther

*R. Soc. open sci*. **4**, 170833. (Published 25 October 2017). (doi:10.1098/rsos.170833)

In our work, we reanalysed phylogenetic datasets for dinosaurs. This included a dataset assembled by Langer *et al*. [1] which was further modified by Baron *et al.* [2]. The dataset analysed in our paper incorporated the modifications made by Baron *et al.* [2] to the coding of multiple characters in *Pisanosaurus*, which was not made explicit in our manuscript (results shown in fig. 5). Also, owing to an error prior to publication, the full citation to Langer *et al*. [1] was not included in our paper and is provided here for clarity. Furthermore, we would like to apologize for the fact that our paper was made available online prior to the publication of Langer *et al*. [1], which was the result of a lack of communication with the journal on our part.
