# Supplementary material for: Multiple optimality criteria support Ornithoscelida
Source: R Soc Open Sci. 2017 Oct 25;4(10):170833. doi: 10.1098/rsos.170833 (PMC5666269; doi:10.1098/rsos.170833)
Supplement: Support for Ornithoscelida: additional information on the methods and results. [file rsos170833supp1.docx]

**Supplementary material**

1. **Additional figures**

**1.1 Bayesian analyses**


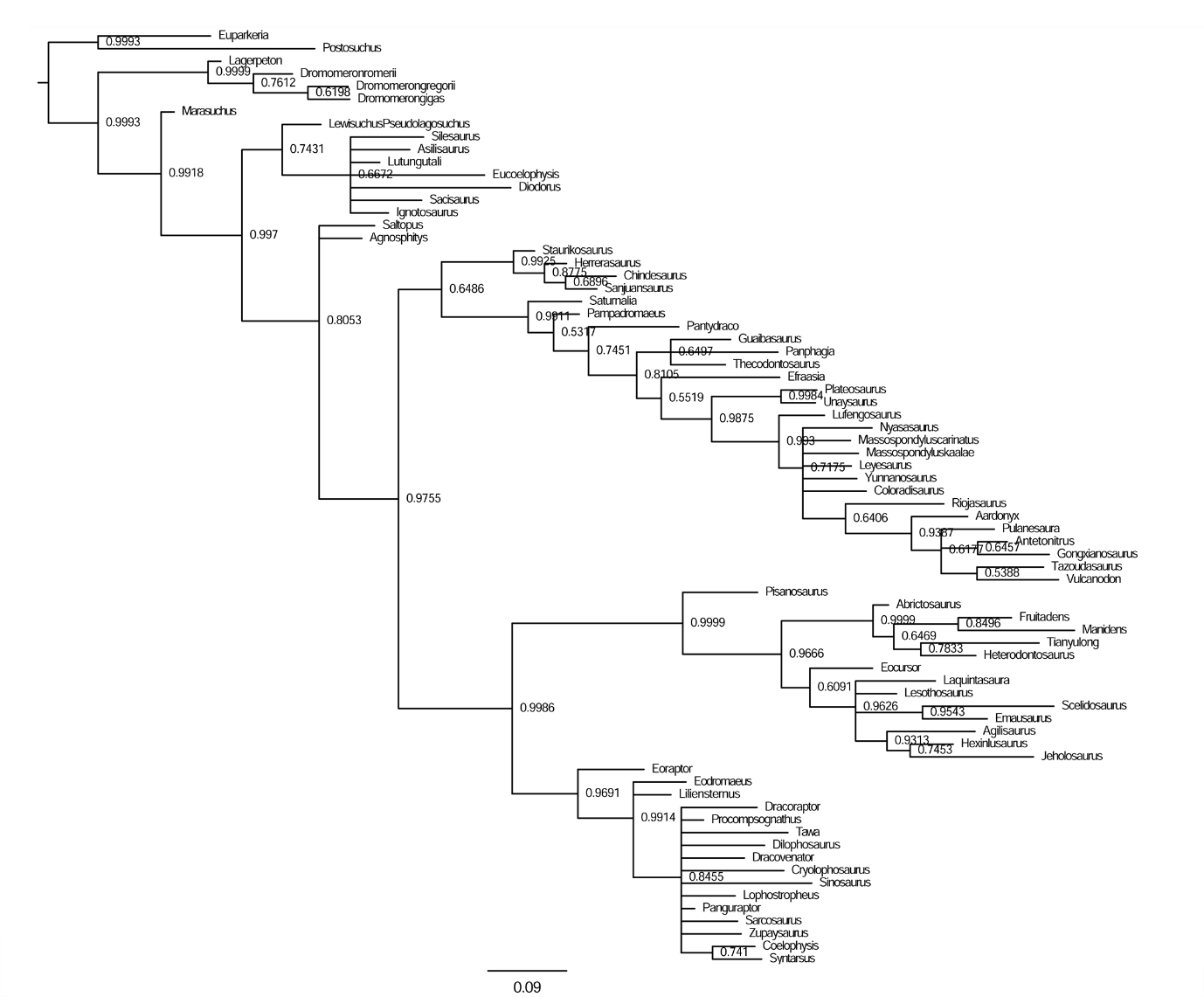


Figure S1. Results of the Bayesian analysis using ordered characters.


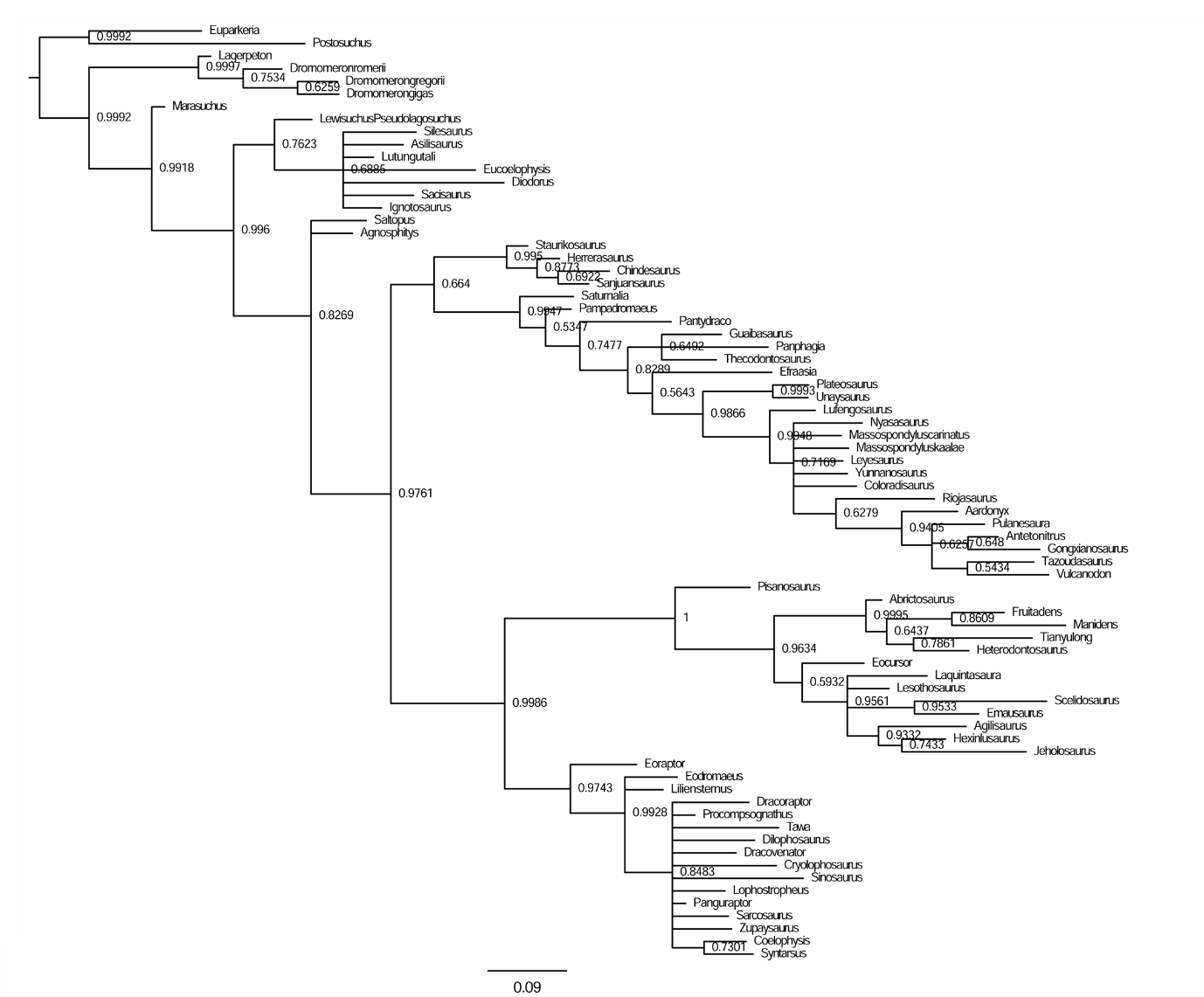


Figure S2. Results of the Bayesian analysis that treated no characters as ordered.


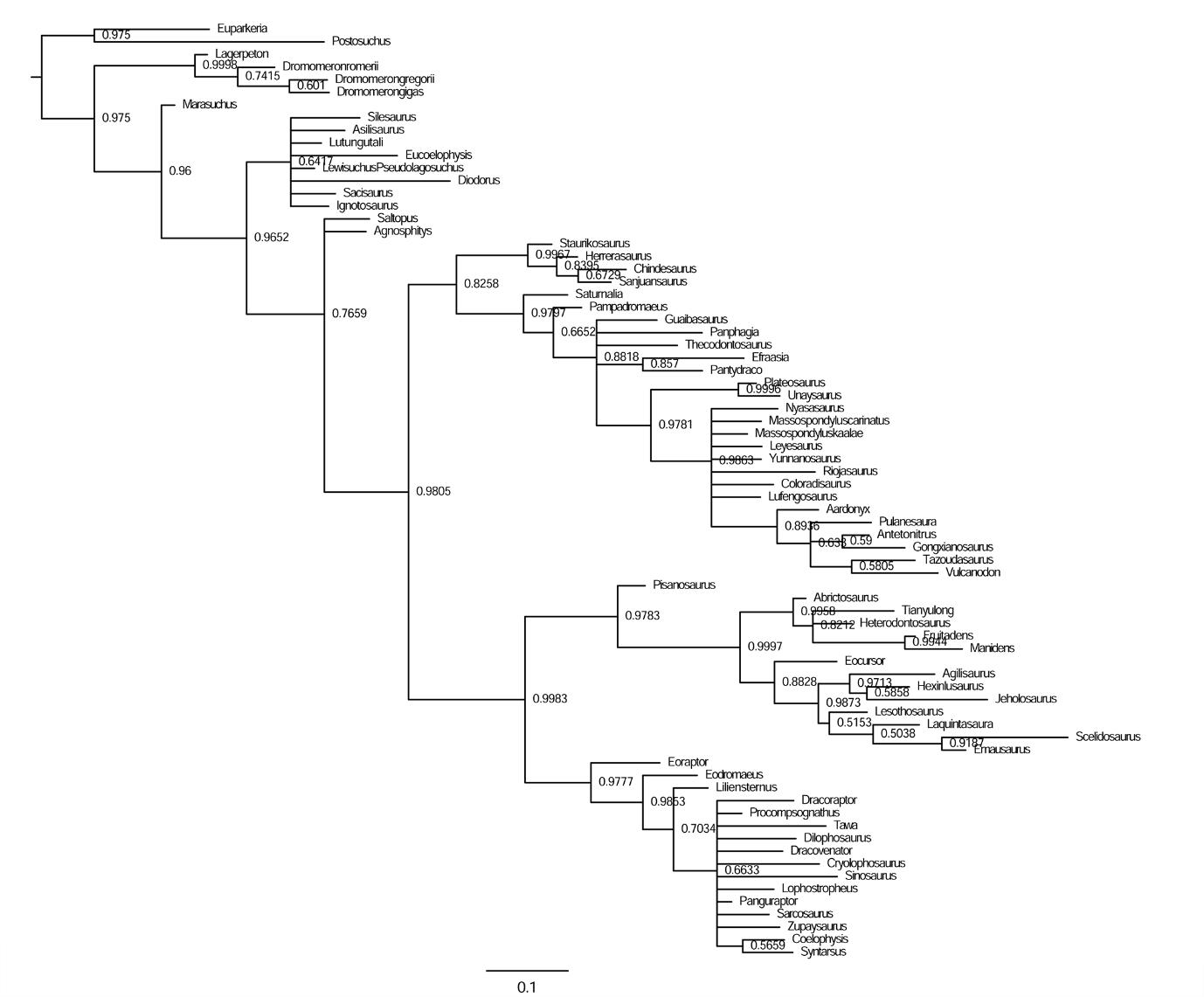


Figure S3. Results of the Bayesian analysis that excluded dental characters.


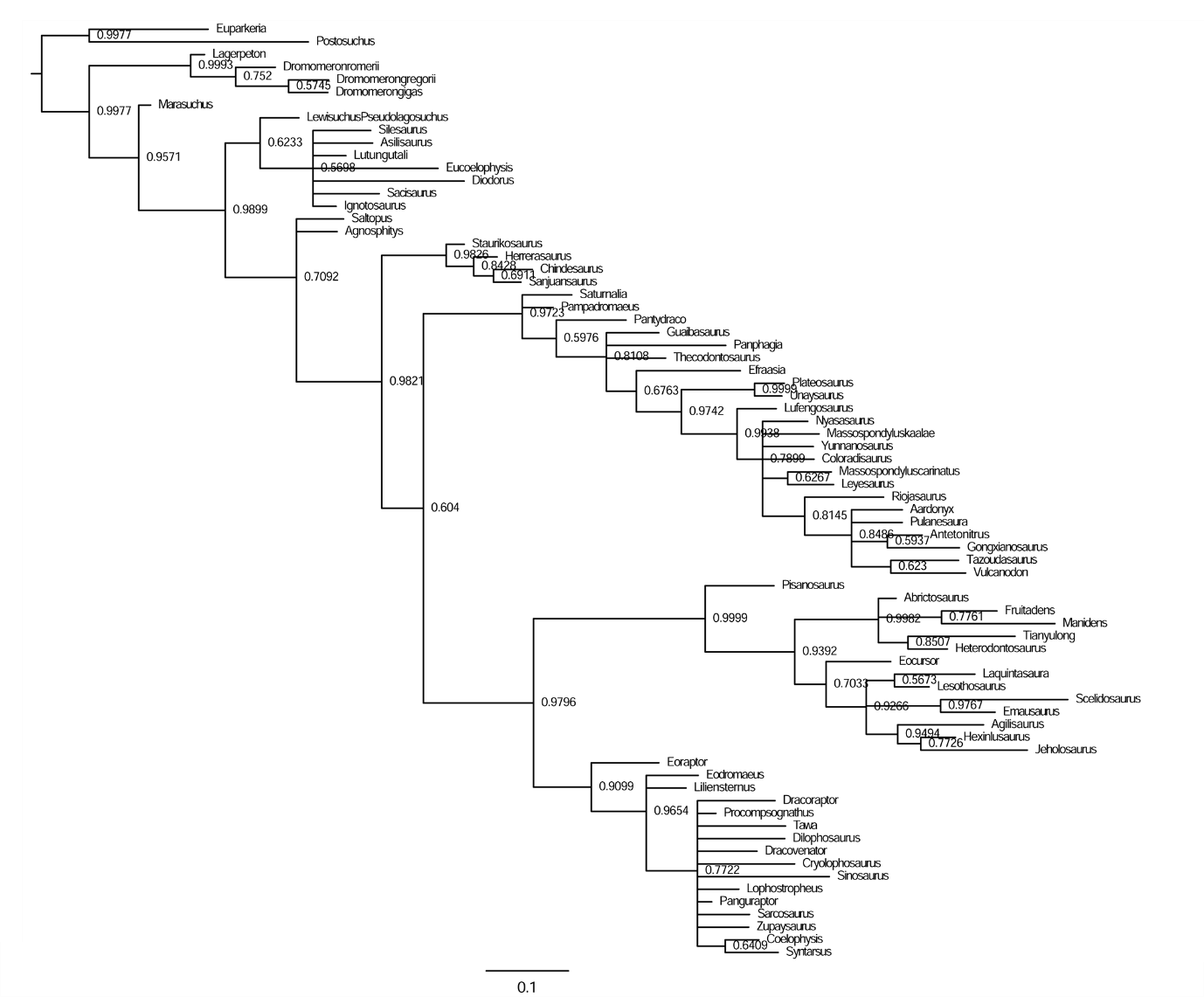


Figure S4. Results of the Bayesian analysis that used no novel characters.


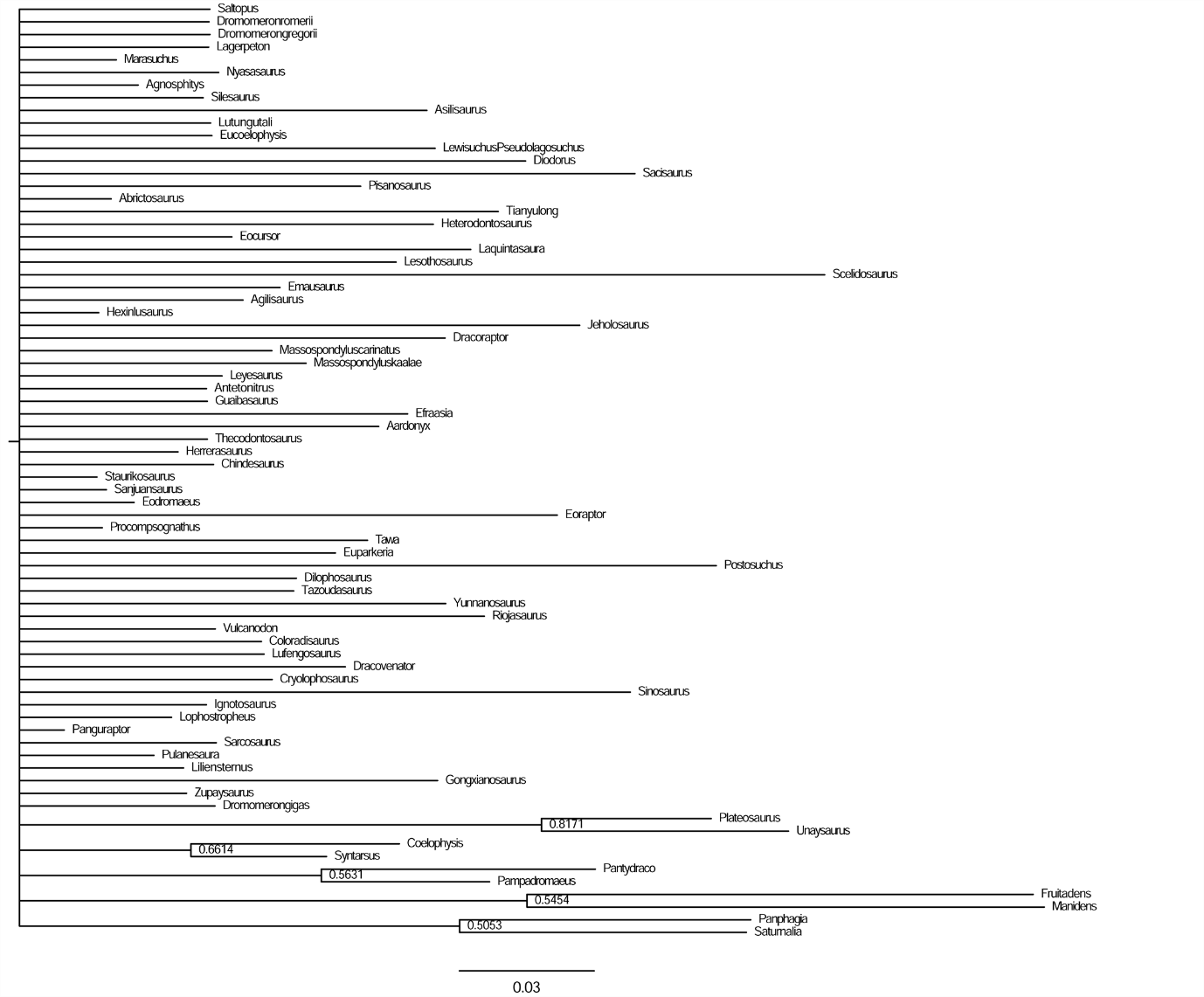


Figure S5. Results of the Bayesian analysis that used no postcranial characters.


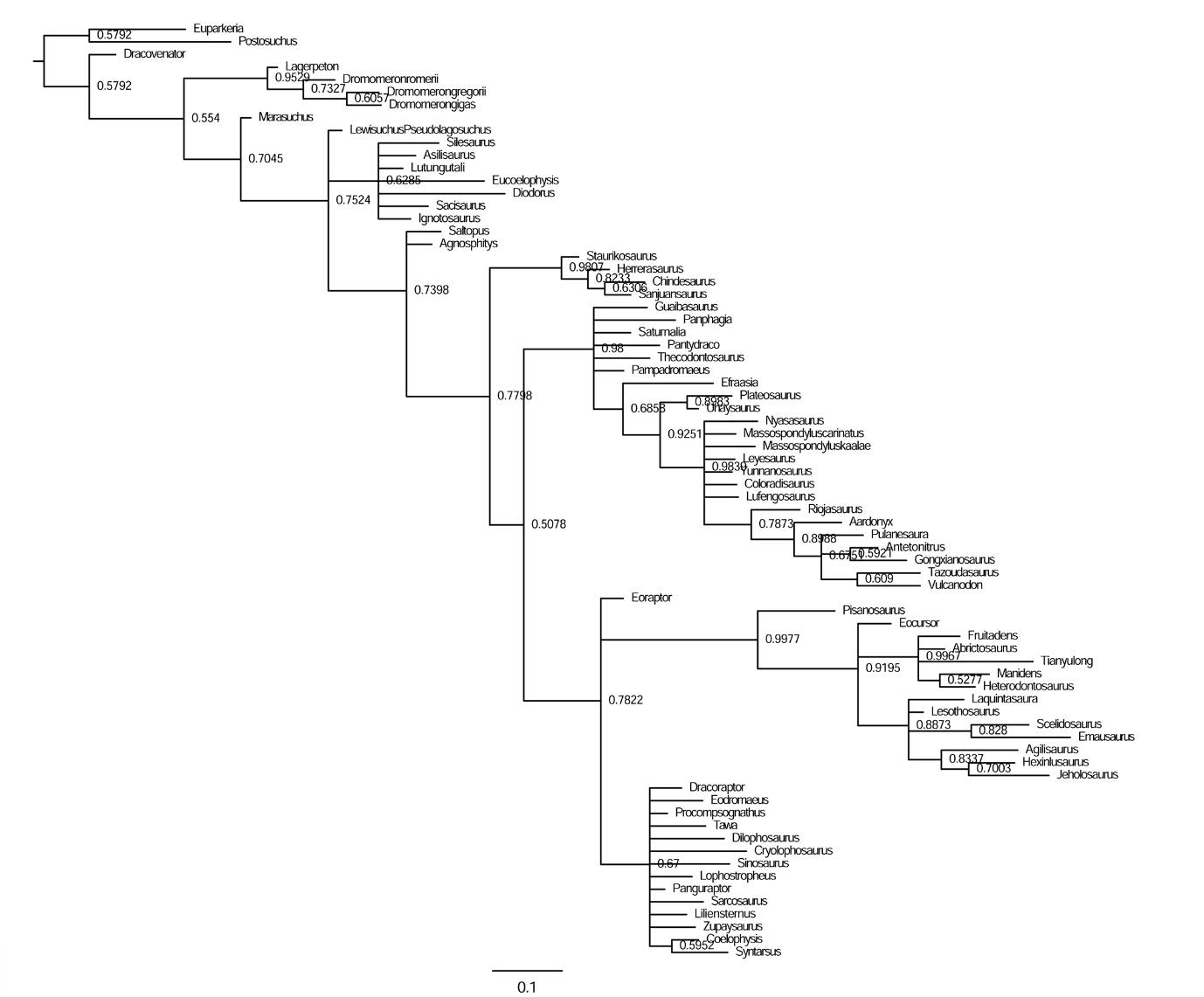


Figure S6. Results of the Bayesian analysis that used no skull or braincase characters.


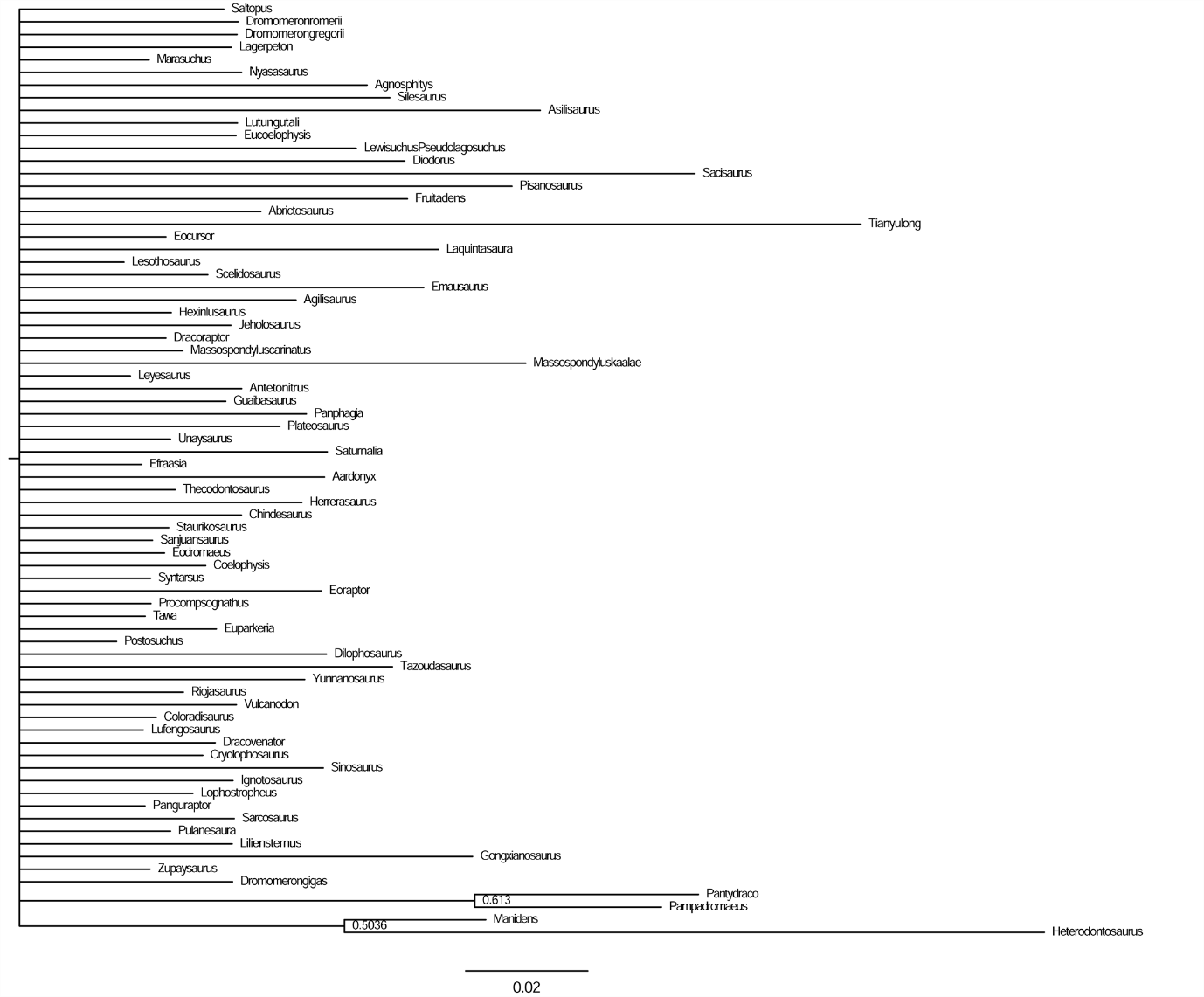


Figure S7. Results of the Bayesian analysis that used only dental characters.


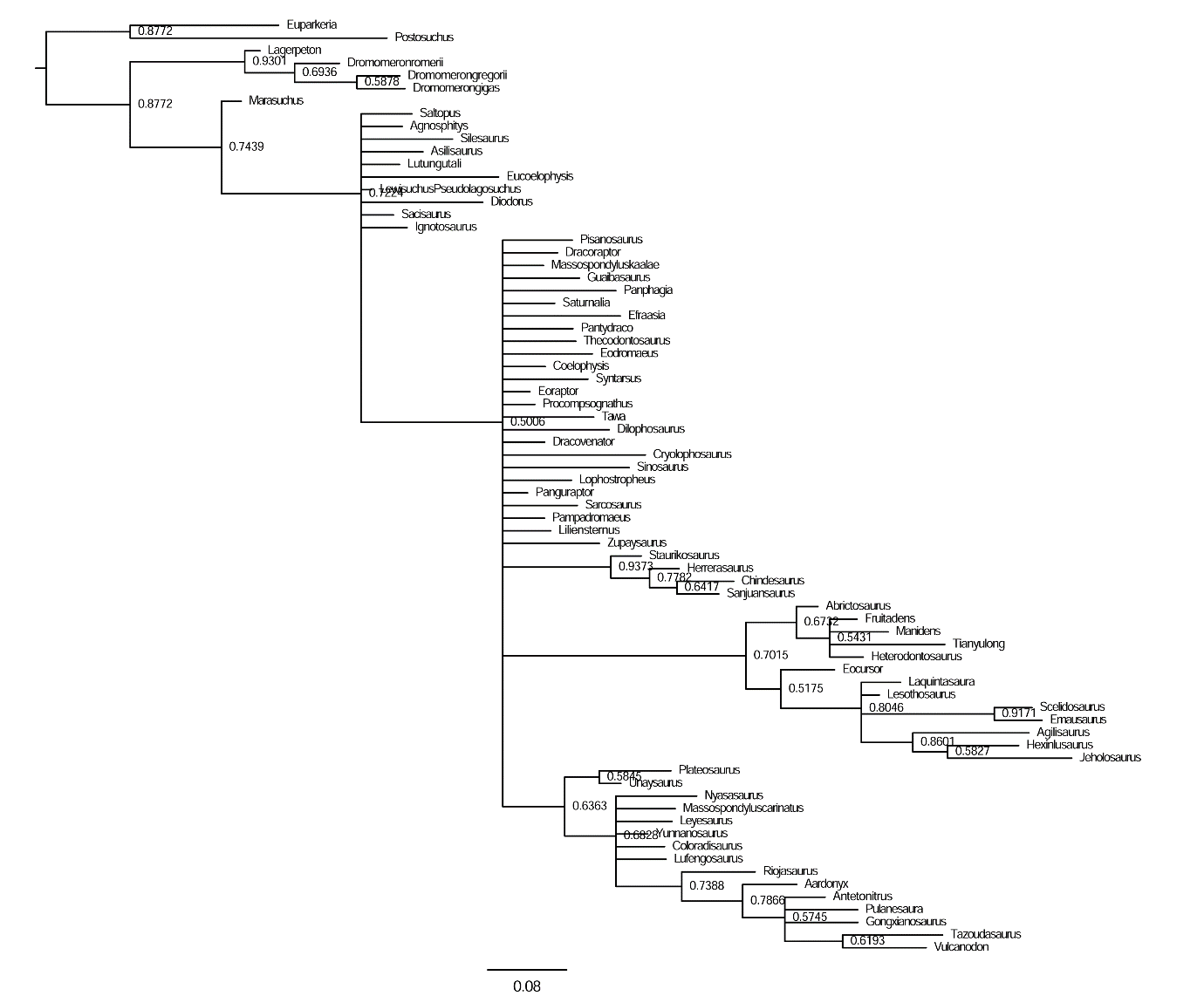


Figure S8. Results of the Bayesian analysis that used only postcranial characters.


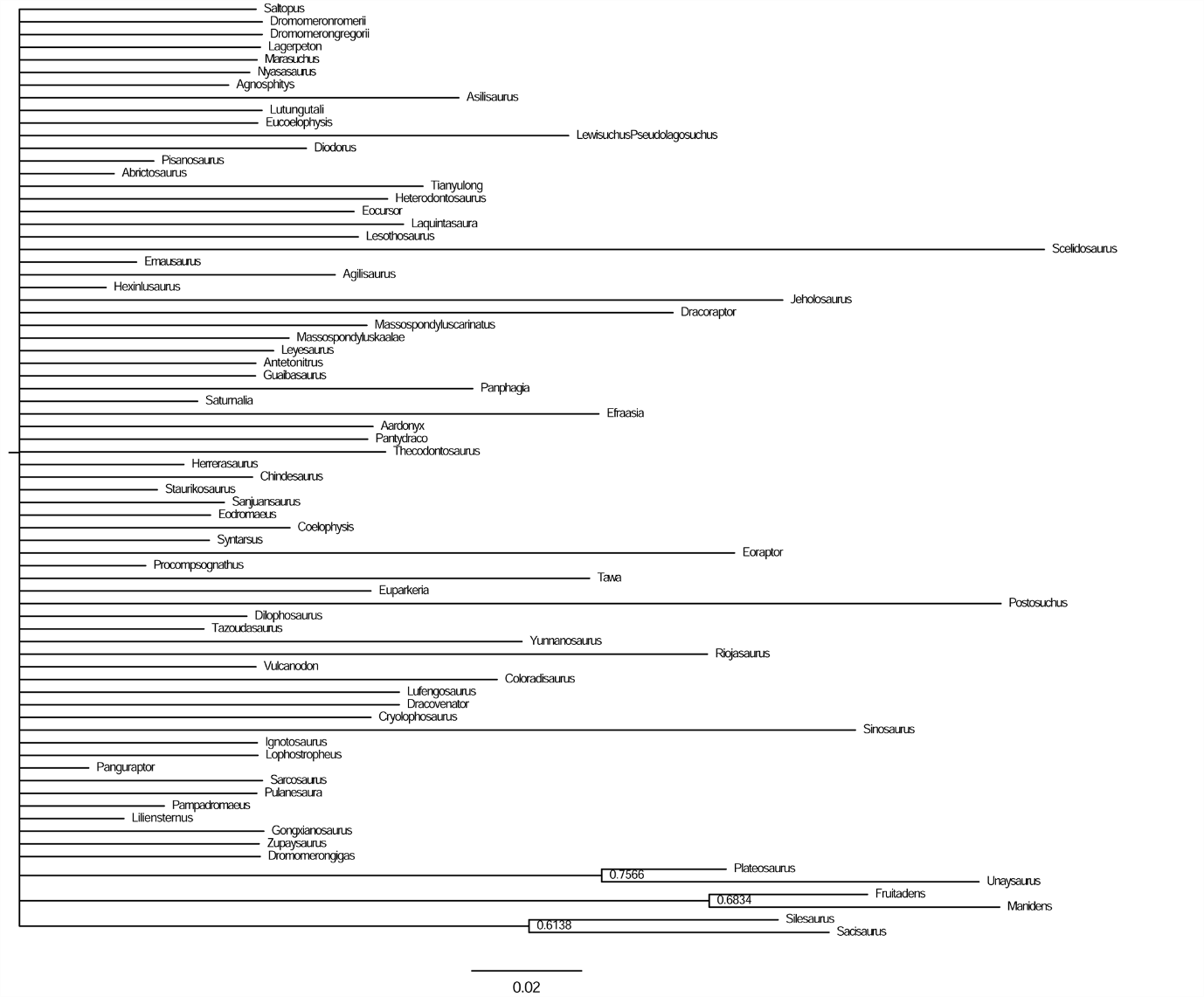


Figure S9. Results of the Bayesian analysis that used only skull and braincase characters and no dental characters.

**1.2 Parsimony analyses**

**
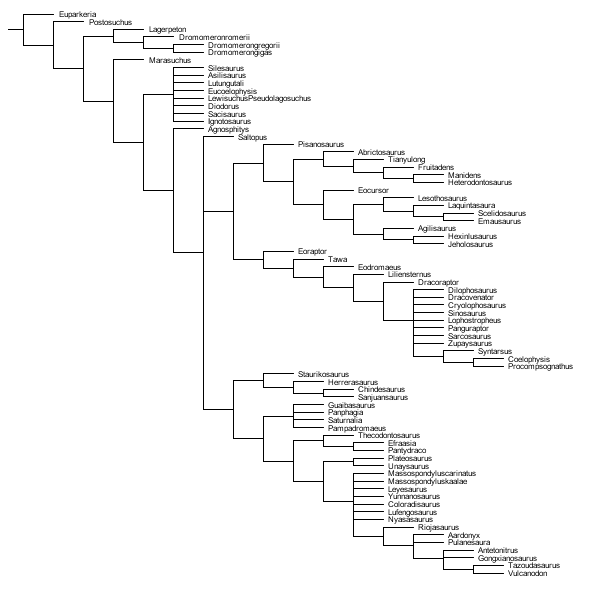
**

**Figure S10.** Strict consensus tree produced in the implied weights parsimony analyses that used the dataset of Baron et al. 2017.

**
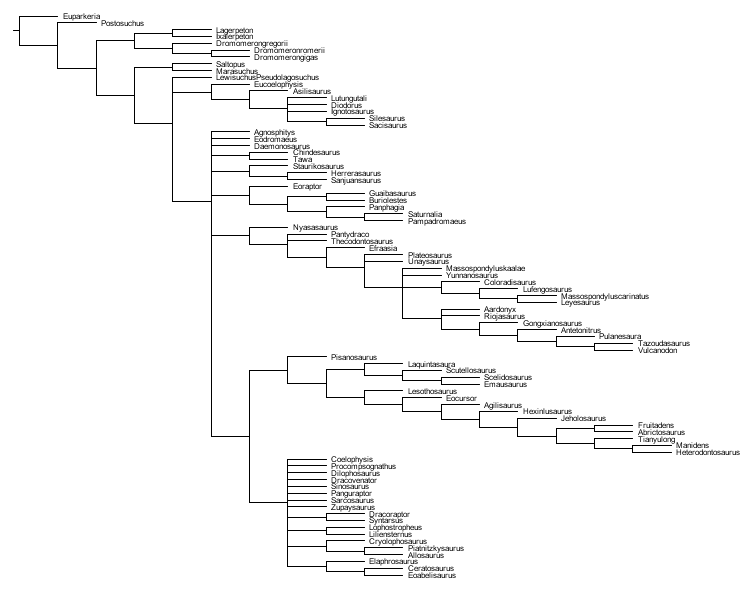
**

**Figure S11.** Strict consensus tree produced in the implied weights parsimony analyses that used the dataset as modified by Langer at al. 2017, when k = 3.


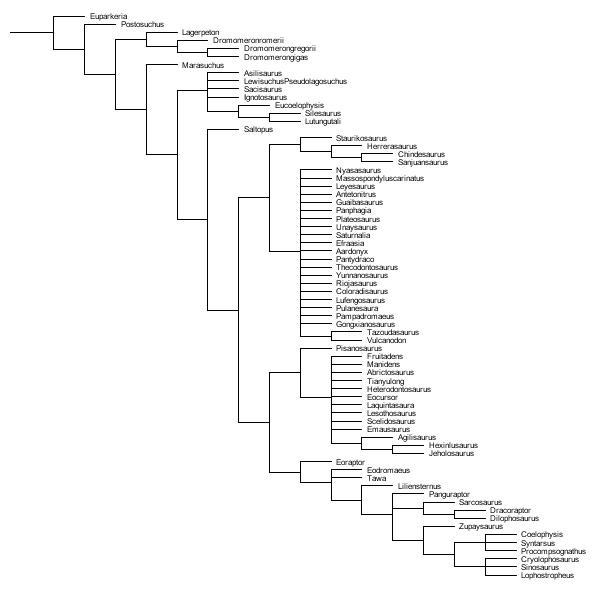


**Figure S12.** Reduced strict consensus of the equal weights parsimony analysis which excluded postcranial characters. The wildcard taxa *Agnosphitys*, *Saltopus*, *Diodorus* and *Massospondylus kaalae* have been removed *a posteriori*.


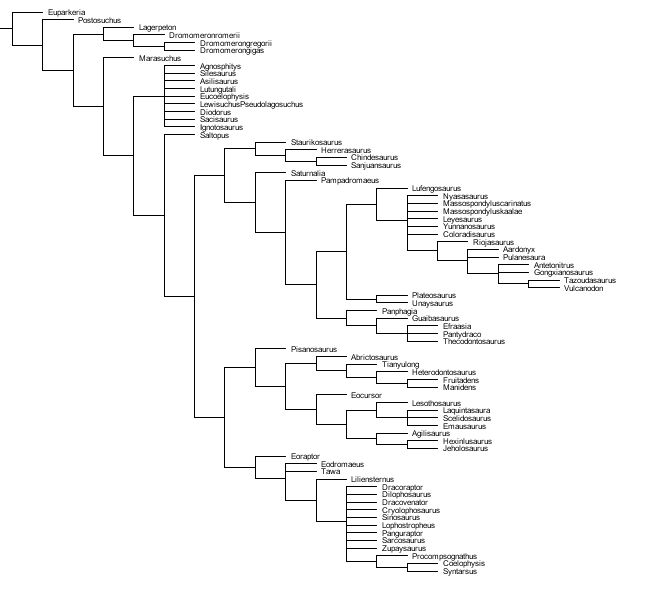


**Figure S13.** Results of the equal weights parsimony analysis that did not include dental characters.

**
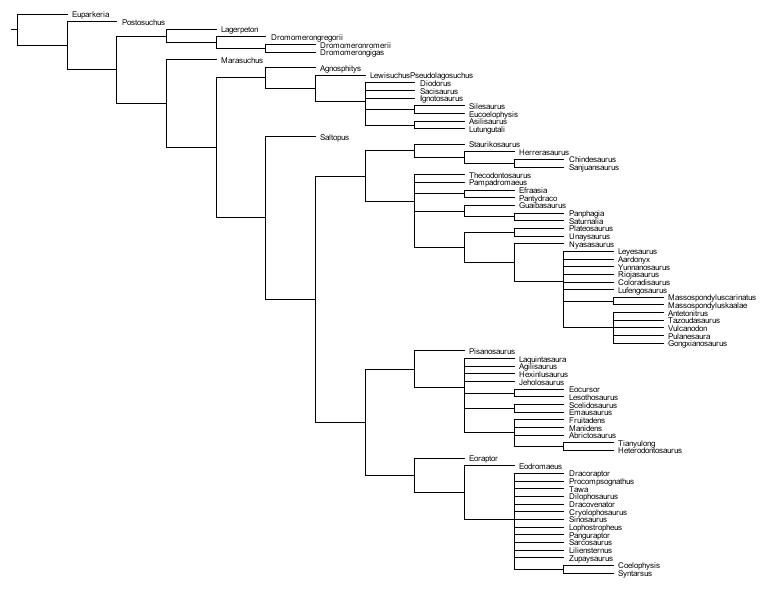
**

**Figure S14.** Results of further implied weights parsimony analyses that used the function xpiwe on the dataset of Baron *et al*. 2017. For the analysis that produced this particular tree, k = 5.


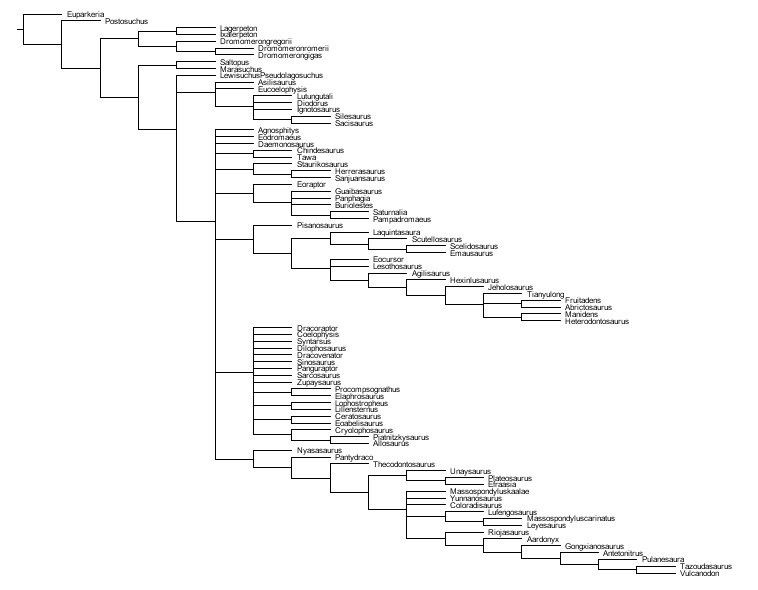


**Figure S15.** Results of further implied weights parsimony analyses that used the function xpiwe on the dataset modified by Langer *et al*. 2017. For the analysis that produced this particular tree, k = 5.

**2.0 Implied weights parsimony**

**Table S2.1 results of the Implied-weights parsimony analyses.**

| \| **k value** \| **no. of steps** \| **no. of MPTs** \| \| --- \| --- \| --- \| \| 3 \| 177.82 \| 70 \| \| 5 \| 135.10 \| 21 \| \| 10 \| 85.43 \| 25 \| \| 12 \| 74.61 \| 22 \| \| 15 \| 62.72 \| 25 \| \| 30 \| 35.07 \| 27 \| \| 50 \| 22.12 \| 23 \| \| 60 \| 18.67 \| 26 \|   **Original Dataset** |  | \| **k value** \| **no. of steps** \| **no. of MPTs** \| \| --- \| --- \| --- \| \| 3 \| 191.76 \| 39 \| \| 5 \| 148.35 \| 38 \| \| 10 \| 95.94 \| 90 \| \| 12 \| 84.19 \| 89 \| \| 15 \| 71.18 \| 66 \| \| 30 \| 40.28 \| 64 \| \| 50 \| 25.55 \| 65 \| \| 60 \| 21.61 \| 55 \|   **Modified Dataset** |
| --- | --- | --- | --- | --- | --- | --- | --- | --- | --- | --- | --- | --- | --- | --- | --- | --- | --- | --- | --- | --- | --- | --- | --- | --- | --- | --- | --- | --- | --- | --- | --- | --- | --- | --- | --- | --- | --- | --- | --- | --- | --- | --- | --- | --- | --- | --- | --- | --- | --- | --- | --- | --- | --- | --- | --- | --- |
|  |  |  |

1. **Character scores**

Saltopus 0??????????????????????????????????????????????????????????????????????????????????????????????????????????????????????????????????????????????????????????????????????????????????????????????????????????????????????00??0???0?0?????????????????1???????11??1??1?0??0-??10?????????0????????????0?00??????1????????????????01?000221?-?00010?1??02???????????1??????00??????1?0??000??????????????????????????0?????000???0??????10?0??0?????10???????02?10?000?0000000

Dromomeronromerii 0???????????????????????????????????????????????????????????????????????????????????????????????????????????????????????????????????????????????????????????????????????????????????????????????????????????????????????????????????????????????????????????????????????????????????????????????????????????????????????????????????????????????????????????????1111000001110--011-----0-0----0011010000000100-0?010100??011?11000100?01?0????????????????????????????????

Dromomerongregorii 0???????????????????????????????????????????????????????????????????????????????????????????????????????????????????????????????????????????????????????????????????????????????????????????????????????????????????????????????????????????????????????????????????????????????????????????????????????????????????????????????????????????????????????????????11110100011?0--1101-0000-1000-0011010000000000-0?010?????01??????????1????????????????????????????????????

Lagerpeton 0??????????????????????????????????????????????????????????????????????????????????????????????????????????????????????????????????????????????????????????????????????????????????????????????????????????????????00??00??0?00000??????????????????????????????????????????????????????????????????0001000001000000?0000200010100000010-10000111011000---1001-11111000001110--011-----0-1100-0?010100?0000000-0?01?100?00101110001?01010000010011301000002110100000??00??

Marasuchus 0?0????????????????????????????????????????????????????????????????????????????????????0?1?010?0???0?10000?00?????????1?????????????????????????????????????000??0???0?00??0??000?000??00011?00-00?0000010-00-00??010??00000000000000010001?????1??00101?111100100??0011???0????????????????????????100100010100000001000200010110000110-10100011010000---1001-01100010001100--1001-0010-1100-10000100000100010?0000100000100110100000000000011010301?0000210010000000000?

Nyasasaurus 0???????????????????????????????????????????????????????????????????????????????????????????????????????????????????????????????????????????????????????????????????????????????????????????11???0?010?1?1?0??20???????1?0?001?010??????????????????????????11111?01??????????????????????????????????????????????????????????????????????????????????????????????????????????????????????????????????????????????????????????????????????????????????????????????????????

Agnosphitys 0????????????????????????????00101000?02???0????????????????????????????????????????????????????????????????????????????????????????????????????????????????0000-100-?0??10?0?00010????0?0?????????????????????????????0?????00?????????????????????????????0011001?00???????????????????????????????00000?1110102?0010002010??????????????????????????????????0??????????????????????????????????????????????????????????1110101101??????????????????????????????????????

Silesaurus 0??01?0000000-000-0-101?0-00???10?0000?2?00?????0????????0000010???1??????000???01?01?00111110110100?0?110000?1????010100---1001120001?0?????10100010102????10?1?100-100020?0?200101011000?0100-002000001101110000001?01?010010100?1000-001?????11000-0101111000000?0010-??0????00??????????????????00000001110102100000020100101010011?-10102002010100---0001-001201110011011010020000111100-110001101011000100?011101000100010101000010???????10301001002010200000000000

Asilisaurus 0???????????????????????????????????????????????????????????????????????????0???????????????????????????????????????????0---1000010?0??????????000?10???????1000-100-00??0-???2?010?1??000??000-10?0000011??1?0??00?1010000?000100?0000-?01????????????1011?0000???0?0??????????????????????????????000100?????002110000020100?01????1???1??0????0????0---?0?1-001000?10??1010?1001-0?0111000-?10001?000010?0????0?0?111001?0110100000000??????????????1????????????00000?

Lutungutali 0??????????????????????????????????????????????????????????????????????????????????????????????????????????????????????????????????????????????????????????????????????????????????????????????????????????????????????0?????????0?00????????????????????????????????????????????????????????????????00000011101020100000201001010?01??0-1??02??10?0???????????0??????????????????????????????????????????????????????????????????????????????????????????????????????????

Eucoelophysis 0?????????????????????????????????????????????????????????????????????????????????????????????????????????????????????????????????????????????????????????????????????????????????????????????????????????????????????????????????????????????????????????1?????????????????????????????????????????????????????????????????????11???1??????01?010?0?00---???????1200110??2?10010120001111100-01000100100??????????????????????????????????????????????0?????0????????????

LewisuchusPseudolagosuchus 00???????????????????????????001011001?2???0???????????10?20003000?0101-0?000000????01101?1110?101?00100100010?1000??0100---0001001???????????????0001??????0000-?00-00001100?000000001000?1?00-100?00001??11101000110?????????????00????????????0001101011???010?0?????????????????????????????????????????????????????????????????????????0????0???00---00????110?0?100???10?1001-00?111100-?1000100000100010??01010??00100110100??0000??????????????1????????????000000

Diodorus 0????????????????????????????????????1????????????????????????????????????????????????????????????????????????????????????????0102?0??????????????0?0???????1100-100-000020?0?2??1020??011??????????????????????????????????????????????????????????????????1011100000????????????????????????????????????????????????????????????????????????????????????????????2010001?1?1001011-001111000-0100????????????????????????????????????????????????????????????????????????

Sacisaurus 0????????????????????????????0?0-1000002???1?????????????????????????0?????????????????????????????????????????1????????0---10011000???????????????101??????1100-101?11000-10?20010?0??000?????????????????????????????????????????000110????????100111???1?????????????????????????????????????????0000000??10?0200?0000211??1010100??0-???0?0010????0---?????0110010100?101001011-000111000-?10001?01011000100?011?????????????????????????????????????????????0??00000?

Pisanosaurus 0???????????????????????????????????11???????????????????????????????????????????????????????????????????????????????????????????010111???0??0000200????????11?0-000-11???????211112???000??0????0???0001??1??01??001???????????????????????????????????????????????????????????????????????????????????????????????????????????1????2???????????0?????????????0?10?0020101?1??10?20?1111?????1?000?0000??0?0100?0101011001110101?1000011???????1??????1?????0??00????0000

Fruitadens 0????11???????1?11???????????0?????210????????????????????????????????????????????????????????????????????????????????????????0102?0???????????????0010311111100-000-0121201002011011??000???1???1?0000?????0??01????0?3?1???121?1001?100????????????????????1?1111000???????????????????????????????????????????????????????????????????????????????????????????100012010?0???101201010-12011100002000?110?0??11111101111?110111?10111111?????????????????????0??????0000

Manidens 0??????????????????????????0?0?0-0?21002??????????????110??0202001?020011001111021??00??0?0???1?????1????????????????????????????210011?1-002101020?01??????1001101100?2120?1?201111???000???1?1???????????0??0????01??3?111?12??1????????????????????01011?????????????????????????????????????????11121100?01?0?2111000?0?0?0?10?022??-1??11?0101??01?0??????0??????????????????????????????????????????????????????????????????????????????????????????????????????????

Abrictosaurus 0000??1000?110?011100000010?100??00211020??011?10100001????????????????????????????????????????????????????????????????011010101?010?11?0?0????0??000103011010?1100??0122201?02011111??000???00-????????????????????????0?????1??1??????????????0?001?????1111?11??0001?????00???1100?001?0??001???011120100000201211101--000?0????????????????????????????????01??????0?0?????1?020111??1201???????0????1??????1111???1101?????????1???1???????10?11????????01000001?0000

Tianyulong 000010110001?0?0111000000?0?1000-??111?2000011110??????00???0????????????0??????2???????????????????????????????????????11??010??000011?010??00???00010401?210?0-00??0121200?02001020??000???????????????????????????????1????1???001010?10?????????11????1?1???1????????????0???11??1??10000??11??0??????????????????????????011010????-?0?111?????????????????1???0??0?????????????????12011?0?0011???11???????11????111??????????11???10??0??101?1??0?????110?000110001

Heterodontosaurus 000010110001101011100000010110111001110200001110010000100?00200010112001100010002001010001001110?0101100110000??00???0?011010101001011100010000002000003011210110011001212?110200111111100?001?10010000010-00-001?211?030100012001?0??10?01010-1010011?1?11111?1111?001110-100002110010010000001111011120100000201211101--00010010100210-1111100101??0100?11-??01?0??120?0??101100201110-12110?000021000110????1?11110111111??1?1?1?11?11100100110?010110????1100000100000

Eocursor 0????????????????????????????????????1????????????????????????????????????????????0?????????????????01001?00????????????????00110000101?00001100020?????????10?0-0??0?1??20?0?20110????000???????0?00000?0-00-00???????2?????11?01??????????????010111????101101101001???????????????1??1???????????1112?1000012012?110001000?00101002?1-20011?0101?20100?11-??0110001202010101100201010-120111100020?001201011??11110?100??????????????????????1????0???????0?0????1?0000

Laquintasaura 0???101?????????????????????1000-??10??2???????????????10?000??00????0?????0????11????000?????????????????0???????????????????????????????????????????000???10010000-11??20?0?10?10?????00?0???????????????????????11????????????????????0???????000?1????1?????????????????????????????????????????11?2????00??????0??00?0????0?01202?10??111?0?????01?0??????0???00?2?2??0???1?120101??1201?0???1??????2010110??11??????1?1????????11??????????????????????????0????????

Lesothosaurus 00??10101?0011100-1000?0?10?1001?001011200001110010000100?000??0011?100210001000000111000?0??11000?01100100000?001??10?01000001100100010000021100200??0010?01100-000-1110200002011110110?0?001?00???000010-00-0???011??20111011101?01111?0??????01001101?11111111010001110-100??100??00?0100?0001?1?11120100001101220100020001001012020111011100101020100011-1-0110000202020100101201010-120110000020000120101100111??110010101110100?111?0?100100?1101101-??010?0?0100000

Scelidosaurus 000?1??0100?????????0?0?0??01000-0??1101100?101?011?2?010?0010310010100100000000100110000??????????1??????01?10????????1??????????10111?1-0??100021???0110??1000-000-101020000201111???0?0???1???00??????0-???????111?220??1???0???0?????011-0-??00011????1?1111001?10??????????????????????????????1112011000110111010002001?0010100200-?011100101021100011-1-0?1000?00212010010120101??1201?0?00020????2???110?112??11001?1?1??????01???0???0?00101???002110000100101110

Emausaurus 00??1??00??0??100-?000000?0?1000-00010020??0111001010001??0000300010100?00?0?000?00110?????????????0??????00???????????1?0??00110100111?000021000200??0110101100-000-1100200012011000??000???????????????????????????????????????????10-00???????????????????????????????????0???03??????10??0??1?????????????????????????????????????????????????????????????????????????????????????????????????????????????????????????????????????????????????????????????????????11?0

Agilisaurus 00011000000010100-1000000?011000-001110200001110010111100?000030001010??10?01000100?011?0??0??1?00?0??????0??0?0??1????010?001110010111?1-0??0000200010110101000-000-111220000201111011000?0?????11???????????0???????1201?10120?0?0?????0?1-0-?10001101??1000?1101000?0-100????????????????????????1112010100110121010002100?001012020111001100101020101011-1-0110???10?0??1011??20101??1201?100101?????20?01??01?110?100101011??????1???0???00003110?00021100000?0100000

Hexinlusaurus 0?0?1??0???????????????????11000-??11?0200001110010000100?0000?0001010??10?01000?00?1110???????????????????????????????0??????1??00??11???????????0?????????10010000-11??2000?201101???000???0???01????????????0??????120????11??1?0?????0?1-0-?1??01??????1???0?0???????????????00??0??01??????????111201010012?1210101--100?0010122210110111001010?0111111-1-0110????0???????1??2010???1201????00?????????????????????0???1?????????1???????1?????????????1?1?00?0100000

Jeholosaurus 00?01011000010110-10000001011000-00010?200001101010000100?0010300010101-10001111010?11100?02??1000?01????000???????????011100011?01011101-0020?012000?00101011?11000-111?200002011?101100000?1??001000001??0110010211??300010121211001110011-???11?101????1111111?1?00???0-?????????????????????????11120101000201201101--100?0011120211120011001?????1???11-??011?001202020101101211110-1201110000211?1?201011001111011001010110010001?110???11003110000021101000001?0000

Dracoraptor 00??100011??1??1??100?00?10?000??11?0?12????1011?0----?00?1001300??0??????????????????????????1001????????????????????????????????????????????????????030???0000-000-0???10?0000000????0?0???????1???1?0???1?1???0????????????????0??????010111????????????????????????110-??0???????????00??1?????????????????????????????????0102112???????2?010?0??0---?01010?200002?201011010?201011?1?0????????????????????????10110?????????????????0??????0????01002110?0?0???00000

Massospondyluscarinatus 0001010011200-010-1111111-11?000-01000021010011100----10100000010011000001000???111110001010?0100???1101?01010??1?1??2?00---01010110110?000001000200110211200000-000-002021001210100111010???11??01?00011100112??0?11?01?00001?0100?0???001??10?0010110?011012111011001100-103?1100??000001111211?0010010001001?012101000?000?001?20????-???0?001010200---???0100???0??00010?????????????1101-?0????0????1???10000?0??0000111?111?????0???0?????00100?0010?1100?0000000000

Massospondyluskaalae 0????10011100-?10-1111111-1??00??01000020?00111100----1???0000??0011?0???????????1?1???????01?1?????1??1??110??????????00---01010110111?00001?000200?00210200000-000-??2021001100100?11010????????????????????????????????????????????????????????????????????????????????????????????????????????????????????????????????????????????????????????????????????????????????????????????????????????????????????????????????????????????????????????????????????????????????

Leyesaurus 00??0??011?010?10-111??1?11??00??0100?02???0111100----?0?0000??00?0100??01?000001111???????????????????????01??000?????00---000??010110?0?0011000?00?1021?200000-000-002021?0021010??110?0?0?1101??000021??01?20?0???????????????????1???0?????????????1??1?????????????????????????????????????????????????????????????????????1???22?????????0???????????0??????????????????????????????????????????????????????????????????????????????0110?0???????010?110?0????00????

Antetonitrus 0????????????????????????????????????????????????????????????????????????????????????????????????????????????????????????????????????????????????????????????????????????????????????????????????0???001?10???????201??????????????001??00??????001001????121111101000110111?3????????????1??11?????1?01010?0000012201100???000?1???????????00001010200---0010100100001121200--1011-001101111-0000010000010001000000101000??????????????????????0?1?110??0?1?0?1?00000000?

Guaibasaurus 0????????????????????????????????????????????????????????????????????????????????????????????????????????????????????????????????????????????????????????????????????????????????????????????????????????10?????????1??1????000??1?00????01??????100???0011?????????????????????????????????????????1??????1?0121?2?00000???0?00122002?0-200000020?01?0---00?1-0???????0101?1001?11-001??1101-??0001?????1???100?000???00010101111???00?0?0???0000?01?00102110000000000000

Panphagia 0??????????????????????????0?????????0???1??????00----?????????????????????000011111??????????1011?????????????????????00---000??111110000001100010011??????0000-010100132000?0101001??010???1?0?0?0001011000-00?0011????0?00???1????????????????00011????1?????????????????????????????????????????000?0001?00201100000010000001221?11???2?00001??0??????0001-0????????????????????????????????????0110011001000000?????0?01?1??1?1?0??????????0?????0??????0???0?00?????

Plateosaurus 0000000011100-000-1000111-110010-11011021110111100----00100000301010100000100???0111101010?011?011?001001000000000?012?00---010101100101000011000100110110100000-01000010200002111011110000001101020000111?11100?00111110000010010000110?01?????1000010001101111101?101110-10201201??00010101111110010110001001001221110020101001021221102??00001010200---00001001000?2020101001011-00?111111-100002?000011?0100?010101000111011111100010?01001000101?01101100000000000000

Unaysaurus 00??00?0111010?00-100011101?0020-1111102?110111?00----0?1???00301010100001101??????111???????????1?0010010000??????????00---01???110010?0000?1001100110210100000-000-001020000211100???000?0????????????????????????1?????????????????10?010????00000100011?11111010101100-1?2???????????010?11?1????????????????????????????????????????????????????????????????????????????????????????????????????????11001000?11???????11011111100???????????????001?0?1?0?0?000000000

Saturnalia 0??1????????????????????????????????????????1??1????????????????????????????????????1?0?????????????????0??0????????????????????????????0???????????????????10???0?????132????????0?????10????1??02?000011?00-0010011001?00000001100??10?0??????00001100011011110010101120-1????????????????????????000????1??01012?00000?0001001221?2?00?200200101?200---?0?0101100102000101001001-000111101-1000020000111001000000101000110011?1?100010?011000103010011020?0?000?000000?

Efraasia 00??100011100-?00-1111111-10?010-01001021000?1?100----0???????????1??0????1100001101?0111?????1?0????10?1??????????????00---000??010110?0001-?00120011021020000??000-??102?000210100???000??????0?1???01???0??1?1?10101?10??0000??001???10101???0?0001?0??111111001010?10??1010?200??000?01111111100100100100012012101000?000?001?10011?-11100?01010200---00?1-0010000201010???1011-000??1101-?0000???0001?0?10?????10?0001??110????00????0110?00?10100??02110?00010000000

Aardonyx 00?101?010200-??0-1010101-1??0?0-0100002???0?????0----0??0000??00??1?0???????????00110?????????????????????????????????00---010??110?10?0??0100???00??011???100??0?????1????0121010????0?0???1?0?0??00011110112??0??1??1???0??1??0?0011??01??1????????????1???????????11011??2???????????01??12?????????????????????????????????122???????2?000?10??2?0---000010?10?00201120???1011-000??1111-00000??????1??01??????????00???????????????????????0??1??0?????0???01?00000?

Pantydraco 00??1?00???00-????101????-10?01????000?21000110100----00?0000??00010?0????0?0???2101100110001?1011?001???0000??0??1????00---000??100?10?0??1-0000200000210100000-000-??12200?0100?011??010???111102?01?0?1000-0110???????????????????010101????????????0??1???01??1?????????????????????????????????10010??100100?200100?2000?00?2202?01-11????????????????????0???????01????????????????11?1-??000200?0?1??01000000??0000???????????????????????0101000102110000010000000

Thecodontosaurus 0????????????????????????????????????1??????????0?????????????????????????????????????0010?0?11001?1?10000000????????1??0---000??110??????????????0011??????0000-010100??2000?210100???000??0????0??0000?1000-?0????????0??0?0?01???10101010????000001000?1?011110110010-0-??00?0001-001?010112111001???????????????????????0????????????????2????????????00???0?10?00201020????0????????1101-1000010000011001000000?????0?00011???100????????????????0??????0???01000000?

Herrerasaurus 000000000000??000-1110000?110010-00000020000110100----000?000030001000000000000010010100101??11001?10100000000?0010????00---000??010010?010010000000000200100000-000-00001001000000011100010?11110?0000011011100??211010000000001010011100???????00111????1??1010110?011110?0000?101-101100010101211100000011000012101000100011012110210022010001010200---0000000101002000101101001-000101101-1010020010010001000000101000110010110100010?01100010301001101100000010000000

Chindesaurus 0????????????????????????????????????????????????????????????????????????????????????????????????????????????????????????????????????????????????????????????????????????????????????????????????0????0????????????????????00???1????1??????????????????????????????????????????????????????????????1???????????????0??0?1???1????????????????????1??????????????100002000101101001-0000?1101-10?001001?01000110?010?????0?10?1?11???0????????????????????????????????????

Staurikosaurus 0???????????????????????????????????????????????????????????????????????????????????????????????????????????????????????0---00?00010010?000????000000???????000???????0001????00000????0?0???1???0??0000???1???0??00101000000000100?00110??????????1????????????????????????????????????????????????101000?1?0?0012?0100010001111?1??21???1?000010?02?0---0000001?000020001011?1001-0001?1101-10?0?20010010001000000101000??????????????????????????????????????????00????

Sanjuansaurus 0?????????????????????????1??0???0000??????0????????????????????????0???????????????????????????????????????????????????????????????????????????????????????0000-000-00??10???00000????000???11110100000?10111001?11101000?0000010100???0????????1011100011???????????11110??????????????0??????????1??????????????????0????????????????????00?01010100---???000010000200010?????0?????101101-1010011010010001000000??10001??01111000?000?????????????0?????????????00????

Eodromaeus 0?????0????0??????????????0??0?1010?0?02??01????0?????1???100?????10???????01??????????????????????01??11?000??????????00---00???01?0?0?0??????01200000201000000-00??0?101000000000000?000?001111021010111010?10???01?01?????01??00???10?0101???010011010?101??11?????1110-10000?100110?1000010112?01??????????1?????1?002????0?1???????????02?02010210---00001?1???0?200010???1?01-?00????????00002?11??????1???0????1000101?1??0?0?0001????0?????????0???????0????000000

Coelophysis 00001000012210??11101101110??000-00100020101100100----1?101001300010100000001???01111111?1?011100????10111?????001???0?00---0000?001010?000020000100100201000000-000-001010000000001011000?111111021111111010?01?0011?0201101110?1010011?010111?100011?101111111111001?110-100001101-11110000101121011000001?00211201110120101001120021?-10101002020200---00101112000?20202011?11020101121????10000211111111011110111011011??0101010110111?010??10311?11002110100000000000

Syntarsus 00?01000012210??111011011100?001?11100020?011?0110----1?1?100030001010000000?????1111110?10??1100??????????????????????00---000??00101??000??1000200100200000000-000-001?10?00000001???000??????????1???1??1???1?0???????1???????????????????????000?1?1011????????????????????0?????????0???????11??????????????????????????1????2???????2??2?????0?????????01112000021102011011020101121100-100002011011?1011000101011011???1?1??111?11?0???0???311?1??021?1?0?0?0??000?

Eoraptor 01001000100010?0101010000100?000-00100020101101100----1?10100030001011010000101101?11????????????0??1??1?010?0?0011????00---0001?0011101000020001200110200000000-000-001010000000100000000???111?11?00001101110010001011000?0010???010???0101??0010011?1?1101111111?001100-10000110001011010111113101000?001?001112?1110020101001220021?-2200?001010?00---00?1-01??00??020101001?020100??1101-100002??????0?01?????1???00011101?10?1000??1011?1?103110?1?02110?00000000000

Procompsognathus 00??1??????????????????????000010??100?20?0110?100----10??100??000??100?0??0?????11110??????????????0??1???????????????0??????1??0010???00?????00200????????0000-00??0???10?0?000000?110?0????????2??1???????????1011??????????????01?????????????????0???????????????1110-?00????????????0???0???101??????1????1?2??1101???0????????????????2?0?020?00---000???1??????0?0??1001?0???????1100-?????211???1???11??01????1??????1??????1????0?????10311?1??02110?00000000000

Tawa 00?01000?0?210??1110?101110??000-01000?2????100100----??1?00013000?110?10000???????10?0000?0?110??0?000110000??????1?0100---00000001010?010????00000?00300000000-000-00?010?000000000??0?0?1111?10?1110011?????0?00??????00?00?0?????011???01???010111????1?11111110??11?0-00???21?0?1111000110?12101?????00?001112?11100?010?0?1????2????????0?20?0?00---?0?00012000?20102010010?20001121100-1000020011010001000011001?00111011111000011101100010301??1002110???0??00000?

Euparkeria 0000000000000-000-0-00100-000000-00000010000110000----000100000000100000000000001000010000100010010000?0000000?0000000000---000000000100000000000000000200000000-000-0?0010010000000001000?0000-0000000000-00-00001??1100000000000?0??0-?000010000110-0000011011100?000????000??000???0?0?10?0?00?0000010000000000000001--00?00000000000-000000120?1?00---0001-000100?000?100--000-----0?1000-?00000-000000?00-??0?00001000?000-0?01?000??00000000000?00000100000000001?00

Postosuchus 0000000000020-010-0-10000-10?000-00200020001100000----0000300020000001011110000011100100111020??010010010?0000?000011??00---01000210010000?001000200000200000000-000-0000100000000000110100???0??00??000?100110010?01022?10000?00??00???0???????00000111?11000011100001110-100??000??0000000100010000101010000100010000001010000102100???1100?0021002?0---1000000100000000100--000-----0?1000-000000-000000110-??11001000000100-0?0?0000??0???000010100100?10010000000??0?

Dilophosaurus 00101000111211?11110100110000001010100120101100110----101000013000111001010000001?110110010011?0011??10110?????????0?2?00---000000110101000??1?00200000200100000-000-001010100000001111010?0111110?1120011011100?00010?2?10001100100101000101???00001101011111111010001110-100??212??01110001101121111000001001212211110120101011?11121??00?011020002?0---?0?01112000020101011010120?01121101-00?0020111121?01110110101?00111011101??0?11?00100010211?11002??0100000000000

Tazoudasaurus 0????????????????????????????????????0???????????????????????????????0?????00???0?0?????????????????????????????????????0---01010110?10?0????0000?0010??????00010000-00??21?0?210110???000??0????0??000?1???11????201?????????????1000???0??????????????????????????????????????????????????????????????????????????????????????1???????????00?1?010100---001??0???????121?????1??????1????????????1??????????????????1?00?11111?1?0?0????????????????0??????0?0?11100????

Yunnanosaurus 00?101?001?01???0-1?01?1?0??000???1?0?02000?110?00----1??1?00??000??00010?000????10?100000???00001?????????????????????00---01???1?0?10?0?????????0???0?????0000-000-0?2?2??0?2100001??0??1??1?010??000111?0112??0?01??10000010010100????01????10000??????101011101?101100-??2??1?0??00?001011111?00100100?0?001012011000200000112200201-2??00001010200---0010100???00201010???1011-000??1101-1000020000011001000010101000111111?1?0000101????????10110010111000000000000?

Riojasaurus 0000010?11?0????0-110?011???000???1001?20??011?100----00112000?1000110000?001????11110?????????????????1???????????????00---01?????0110?000??1?00200?101????000??0???0???2????21010?1??0?0?0?11000???00?11001020??1110???11????0???00???0??????????????1011111111010101100-1?201?001-001?0111111131010010000000201201101--000?001?20220?-???00001010200---0011-0010000202110???1011-000101101-1000020100111001000010??1000111?1??????00???10???0?0101?00102?0001????00000?

Vulcanodon 0????????????????????????????????????????????????????????????????????????????????????????????????????????????????????????????????????????????????????????????????????????????????????????????????1?????????????0?????????1??0??0????1????0????????????????????11??????11011?????????????????????????1???????????????1101--001??010?012?1-1?000?11010000---001000?100002121201?01011-001101111-?????10000?????10?????1010001111110?00100?011----?0000110010?10001?01000000?

Coloradisaurus 00??010011201-?10-11011110?01000-01001?2???0????00----??10?0003??011?00001000010110110111010?11001?011?100120????????0?00---010??10011010?001000110011??????00010000-002?21?0?210100???0?0???1?0?0?00001???011201?0111?????????????00???0???????1000110101101111101100???????????0??????????????????1???????????012?010001000000122102???22000001010200---0010000???002020101?01011-001??1101-0000020000111001000010??1??0?111111?0100????0????00030110010110000000000000?

Lufengosaurus 0001?????????????????1??1??2?00???100?020??011??00----1?10?????01001101-01000???11011000?0???11001?01??1?0000??0011????0??????????1?010?0??????0010?????????0000-000-00??20?0?21010?1110?00??10010200001???011101?01110100000100101001100010?0-100000101001011111011001100-1?2111001-00??01011111?001001000100020?211101--00000?12200201-22000001010200---0000000100002020101??10?1-000??1101-1000020000?11001000010101000111111?111?0110?0101101010110010111000000000000?

Dracovenator 0????000102211?11111?101100??0?1001200?2????1???1??????????????????????????????????????????????????????????????????????????????0????????0??????002?0??020?000000-000-000?10?00000001???0?0????????????????????????????????????????????????????????????????????????????????????????????????????????????????????????????????????????????????????????????????????????????????????????????????????????????????????????????????????????????????????????????????????????????????

Cryolophosaurus 00?01??????????????????????0?00????10?1?010?100110----??10100?3?1?1??0??01000000?011??100100100000??????1?0?????0?0??0?0????????????????0????10002??????????????????????????0?0000?????0?0????1??0??110??111100?00??1??2????0????1???1???0???????????????????????1?0?01100-???????????????????????????????01?0?10????1100?????001021??????0?02?0?01??00---?0?????20?00200010???1101-000111100-10000??????1??01100??1???10011101?0?1??11111??????????????????????????00????

Sinosaurus 0010100011201??0111111011000000??00?0002??01100110----0???0000100011110201100????011101??1?2?11000?0101110000????????0?00---0001000?010?000??0000200??0101000000-000-0010100000000001??000???????1??11?????1??0??0??1?12?1??0?????0?0????01??1??10001101011111111?1?00111??1????????????????????????11030001?0121120011001010?001?20020?-1?10?00201020????01-???020?0??010???1?1??2010???1100-0?1?021111???101?0?01?1000001???1?????1101110?????103????0?0211?201?0?000000

Ignotosaurus 0???????????????????????????????????????????????????????????????????????????????????????????????????????????????????????????????????????????????????????????????????????????????????????????????????????????????????????????????????????????????????????????????????????????????????????????????????000100011101120000000201001????????????????????????????????0??????????????????????????????????????????????????????????????????????????????????????????????????????????

Lophostropheus 0???????????????????????????????????????????????????????????????????????????????????????????????????????????????????????????????????????????????????????????00???????????10???00?????????????1???0??1111111010?????????2??1?010??1??1???????????????????????????????????????????????????????????????11?0?0?1???21?2?0110?2010?0???????????????????????????????????????????????????????????????????????????????????????????????????????????????????????????????????????????

Panguraptor 00?01???????????11??????????0001010100?20??110?100----??1?10013000?0100100001??????110?????????????????????????????????00---00???001??0?00???1???20000??????000??0???0??01?00?000000???000??????1?21?1111??1??0??0?01?0??????????????????????????10011????1??????????????????????????11??0??0??111??1?????????????2??1?0??0??????02??????10?????????????????????1?????2??0?0???1?0???00????????????2????1????11????????1?01?????????00???10???01??????1?00?1?0?0????0?000?

Sarcosaurus 0????????????????????????????????????????????????????????????????????????????????????????????????????????????????????????????????????????????????????????????????????????????????????????????????????????10?????????????????????????????????????????????????????????????????????????????????????????110300010002012011101201010?1???????????02?0??00?00---?????0???????010??11?1001-?00??1101-????????????????????????????????????????????????????????????????????????????

Pulanesaura 0???????????????????????????????????????????????????????????????????????????????????????????????????????????????????????????????????????????????????????????00010000-00??21???21?10??????0??0100???00002?1001111??101??????????????101??00???10???????????????11???????1011??????0??????????????????????????????????????????0??0122002?1-??????????????????????0???????????????????????????????????100?001??0100?000?????????????????????????????????????????????01?00000?

Pampadromaeus 00?010001120?0?00-1??1011?1?0010-1100002000?110100----0?0?0000300010?0????000????0??10?????????????????????????????????00---000??000010?0??0?0?00100100210100000-000-0?1320000100100101010???????????????1???????0????????????????????????????????0001????1?????????1????????????????????????????????001?00000?10?2?010002000?0????????????????????????????????0???0???0001?1001?01-00?111101-?????1??0?????????????????????????????????????????????????????????????0?0000

Liliensternus 0??????????????????????????????100?1?0?2???????????????????00?30?0??10010?00????????01?????????????????????????0????????0---00???011???????????????0?0??????000??0?????????0??000100???000????11???1?1?1?1?1??0?????1?????????????????????????????00???1??1?0111111?011110-?????????????????????????110000?1?011??2011?012010?000?20?21?-???0?002010?00---???010????0??020?010?1??201001?1101-10000?01??1????11????????000???????????????????????0?????10????0?000?00?????

Gongxianosaurus 0?????????????????????????????????????????????????????????????????????????????????????????????????????????????????????????????????????????????????????0?????0?01?0???00??0-????1?????????????????????????????????1?0?????1?????????00110?010???100?00-01??11111110????10-??1??????????????????????????????????????????????0?0????????????????0????????????00???00??00?0121?0???00?-----??????????????????1???100???0???100111?1??????00???0?????00101?0??0?1000100000000??

Zupaysaurus 00??1???????????11????????000001000100120001100110----10100001300010100200001????11100100?????1010??????????????0?1????00---00???011010?000???000200?0??????000??000-????1?0??0000000??000?0???????????????????????????????????????????????????????????1?11??????????????????????????????0????????????????????????????????????????????????????????????????????????????????????????????????????10000??????11001101?11???1001001111000010111????????????????????????????????

Dromomerongigas 0????????????????????????????????????????????????????????????????????????????????????????????????????????????????????????????????????????????????????????????????????????????????????????????????????????????????????????????????????????????????????????????????????????????????????????????????????????????????????????????????????????????????????????????????111010???110--1111-0000?0----?01?0???????????????????????????????????????????????????????????????????????
